# Narrowing the Kinetic Gap Between Alkaline and Acidic Hydrogen Oxidation Reactions Through Intermediate Behaviors Regulated on D‐p Hybridized Pd‐Based Catalysts

**DOI:** 10.1002/advs.202513616

**Published:** 2025-10-14

**Authors:** Lixin Su, Hao Wu, Shengnan Zhou, Runjie Qian, Chenxi Cui, Shaokun Zhang, Liqing Wu, Wenting Li, Huan Pang

**Affiliations:** ^1^ School of Chemistry and Materials Yangzhou University Yangzhou Jiangsu P. R. China 225009; ^2^ School of Environmental Science Nanjing Xiaozhuang University Nanjing Jiangsu 211171 P. R. China; ^3^ School of Chemistry and Chemical Engineering Chongqing University of Science and Technology Chongqing 401331 P. R. China; ^4^ School of Physics and Optoelectronic Engineering Hainan University Haikou 570228 P. R. China

**Keywords:** hydrogen oxidation reaction, acid–base kinetic gap, intermediate behaviors, d‐p hybridized Pd‐based catalysts

## Abstract

The kinetics of hydrogen oxidation reaction (HOR) decline by orders of magnitude when varying from acidic to alkaline environments. Consequently, narrowing the kinetic gap between alkaline and acidic HOR (acid–base kinetic gap) and identifying the prominent reaction intermediates under varying pH conditions remain significant yet challenging. Herein, through introducing p‐block Ga and Sn elements, the HOR behaviors of the constructed d‐p hybridized Pd‐based catalysts are investigated under different electrolyte pH, particularly in alkaline and acidic conditions. Remarkably, the specific activity of PdGa/C alloy surpasses that of other prepared Pd‐based catalysts in both alkaline and acidic electrolytes. Additionally, the PdGa/C alloy exhibits the smallest acid–base kinetic gap among these catalysts. Combining experimental results and theoretical calculations, it is determined that the incorporation of Ga optimizes the electronic structure of Pd via d‐p orbital hybridization, thereby enhancing the adsorption behaviors of reaction intermediates, facilitating the HOR process, and narrowing the acid–base kinetic gap. More critically, it can be inferred that the decisive reaction species for HOR vary with increasing pH, namely, it transitions from H/H_2_O species in acidic conditions to OH/H_2_O species in alkaline conditions, which accounts for the observed trends in alkaline/acidic activity as well as acid–base kinetic gap.

## Graphical abstract

The significantly narrowed kinetic gap between alkaline and acidic hydrogen oxidation reaction (HOR) can be accomplished by the d‐p hybridized PdGa alloy with modulated electronic structure and optimized reaction intermediate behaviors. Additionally, it can be identified that a pH‐dependent shift in dominant intermediates, transitioning from H/H_2_O species in acidic HOR to OH/H_2_O species in alkaline HOR, which is responsible for the kinetic behaviors.

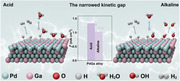

## 1 Introduction

The hydrogen oxidation reaction (HOR), as a pivotal anodic process in fuel cells, is essential for hydrogen energy conversion systems.^[^ ^1^, ^2^ ^]^ Nonetheless, its reaction kinetics is highly dependent on the pH of the electrolyte, with a marked decline in activity (by up to two orders of magnitude) when shifting from acidic to alkaline environments, even for the high‐performance Pt/C catalyst.^[^ ^3^, ^4^, ^5^ ^]^ This kinetic bottleneck critically impedes the commercial development of alkaline exchange membrane fuel cells (AEMFCs), which are regarded as desirable alternative to state‐of‐the‐art proton exchange membrane fuel cell (PEMFC).^[^ ^6^ ^]^ Consequently, advancing highly effective alkaline HOR catalysts and narrowing the kinetic gap between acidic and alkaline HOR are indispensable but remain a formidable challenge.

Significant endeavors have been devoted to elucidating the intricate HOR mechanism, with emphasis on reaction intermediate behaviors and interfacial environment during the HOR process.^[^ ^7^, ^8^, ^9^ ^]^ As is well known, the hydrogen binding energy (HBE) has been traditionally viewed as a pivotal descriptor for HOR activity, with an established volcanic relationship between the current densities and HBE for distinct electrocatalysts.^[^ ^10^, ^11^ ^]^ Nevertheless, this theory faces challenges under alkaline conditions, where HBE alone seems insufficient to adequately describe the reaction process. To address this limitation, Yan et al.^[^ ^12^ ^]^ further proposed the apparent hydrogen binding energy (HBE_app_) theory, which incorporates the influence of interfacial water molecules. On the other hand, Markovic et al.^[^ ^13^ ^]^ demonstrated that the pH‐dependent HOR activity arises from changes in proton acceptor and donor as electrolyte pH increases. Specifically, the acidic HOR pathway involves H_2_O and H_3_O^+^ as the proton acceptor and donor, respectively, whereas the alkaline HOR pathway features OH^−^ and H_2_O as the proton acceptor and donor, respectively. More importantly, they verified that introducing oxophilic species can strengthen the hydroxyl binding energy (OHBE), thus accelerating alkaline HOR kinetics. Additionally, Chen et al.^[^ ^8^ ^]^ highlighted the significance of the catalyst‐electrolyte interface for HOR. Their findings indicate that the OHBE can affect the interfacial water connectivity and regulate the hydrogen‐bond networks within the electric double layer, thereby optimizing the alkaline HOR kinetics. Very recently, through dynamic spectral characterization, Li et al.^[^ ^14^ ^]^ determined that the interaction between interfacial water molecules and adsorbed OH intermediate (OH^*^) on alkaline catalytic surfaces. Notably, it was concluded that the interaction is conducive to forming the weakly hydrogen‐bonded water, consequently enhancing the catalytic process. However, the origin of the pH‐dependent HOR kinetics remains intense debate.^[^ ^15^, ^16^, ^17^ ^]^ Therefore, identifying prominent reaction intermediates under varied pH conditions, especially in typical acidic or alkaline conditions, and understanding their roles in regulating the kinetic gap between alkaline and acidic HOR (acid–base kinetic gap) are significantly desirable but still challenging.

Currently, Pd‐based catalysts, due to their relative abundance and structural similarity to Pt, are regarded as promising alternatives to benchmark Pt‐based catalysts toward HOR.^[^ ^18^, ^19^, ^20^, ^21^ ^]^ Nevertheless, the mechanistic understanding of Pd‐based HOR remains relatively limited compared with Pt‐based catalysts. Furthermore, in comparison to conventional d–d interaction‐based catalysts, incorporating p‐block metals into d‐block metals can induce d‐p orbital hybridization in view of the unfilled and delocalized p orbitals of p‐block elements.^[^ ^22^, ^23^, ^24^ ^]^ Herein, with the introduction of p‐block Ga and Sn elements, the d‐p hybridized Pd‐based catalysts (PdGa/C, PdSn/C alloy, and Pd_2_Ga/C, Pd_2_Sn/C intermetallic compounds) were constructed, and their HOR behaviors under varied electrolyte pH were explored, especially, under alkaline and acidic conditions. Remarkably, the specific activity of PdGa/C alloy exceeds that of other Pd‐based catalysts within this catalytic system in either alkaline or acidic electrolyte. Besides, the PdGa/C alloy exhibits the smallest acid–base kinetic gap among these catalysts. Combined experimental results and theoretical calculation, it is concluded that the incorporation of Ga optimizes the electronic structure of Pd via d‐p orbital hybridization, thereby enhancing the adsorption behaviors of reaction intermediates and facilitating the HOR process. More critically, it can be inferred that the decisive reaction species for HOR vary as pH increases, namely, varying from the H/H_2_O species to the OH/H_2_O species for acidic HOR to alkaline HOR, which is responsible for the observed trends in alkaline/acidic activity as well as acid–base kinetic gap.

## 2 Results and Discussion

**Figure 1:**
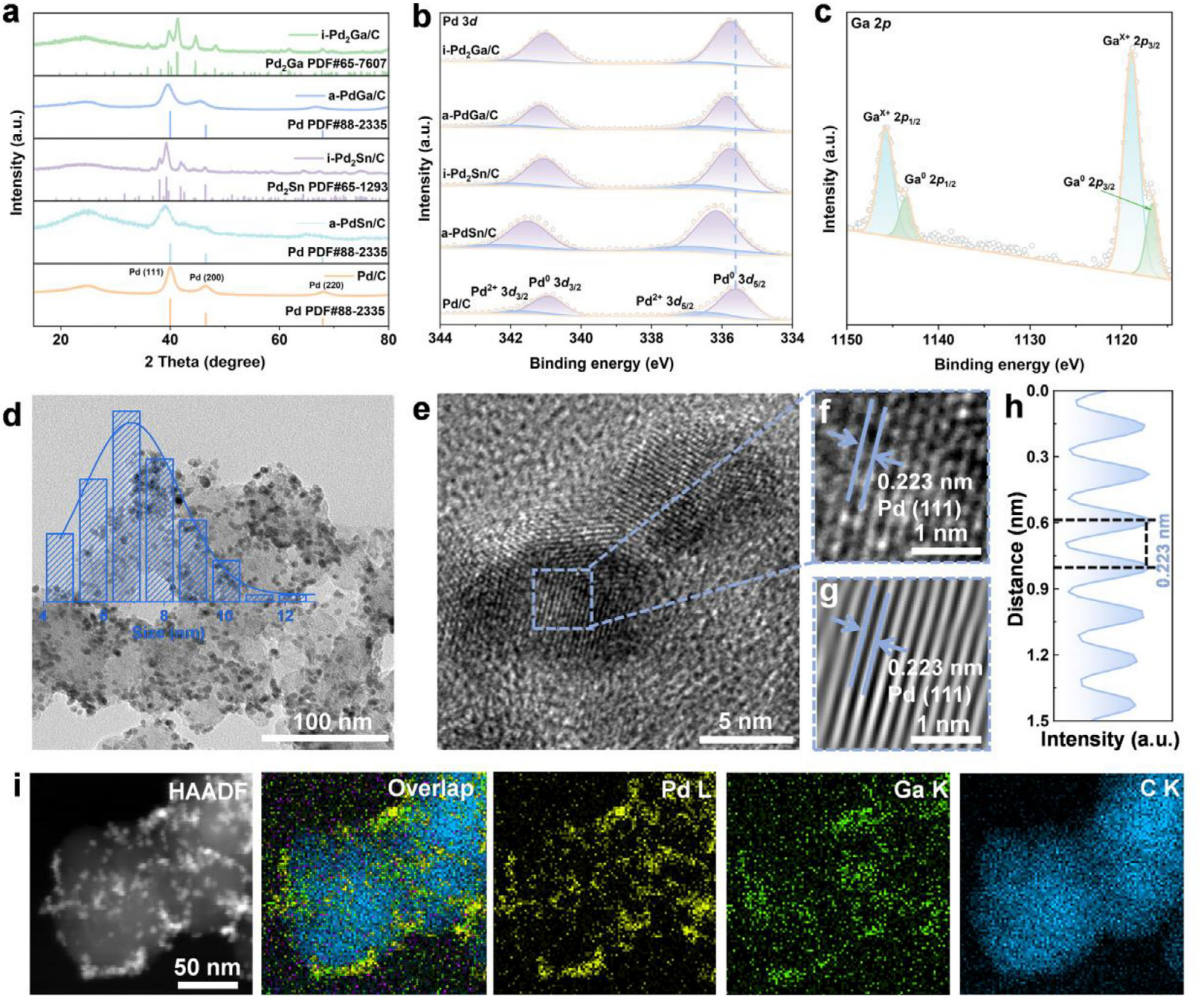
a) XRD patterns, b) High‐resolution XPS of Pd 3d for a‐PdGa/C, i‐Pd_2_Ga/C, a‐PdSn/C, i‐Pd_2_Sn/C, and Pd/C. c) High‐resolution XPS of Ga 2p in a‐PdGa/C. d) TEM image of a‐PdGa/C and the corresponding size distribution histogram. e–g) HRTEM images of a‐PdGa/C. h) Intensity profile of the atomic layers marked by the blue arrows in (f). i) HAADF‐STEM‐EDX elemental mappings of a‐PdGa/C.

The PdGa/C alloy and PdSn/C alloy (named as, a‐PdGa/C and a‐PdSn/C) were prepared by a colloidal synthesis method using palladium acetylacetone, gallium acetylacetonate, tin acetate, oleylamine, and carbon powder as the palladium source, gallium source, tin source, solvent, and support, respectively (see Experimental section for details).^[^ ^25^ ^]^ Similarly, the Pd/C counterpart was prepared based on the above method without the addition of Ga or Sn source. Furthermore, Pd_2_Ga/C and Pd_2_Sn/C intermetallic compound (named as, i‐Pd_2_Ga/C and i‐Pd_2_Sn/C) could be successfully synthesized by the above method with introducing methylamine hydrochloride into the same synthetic system. First, with the characterization of X‐ray diffraction (XRD), as presented in **Figure** **1**a, the structural properties of these materials can be explored.^[^ ^26^, ^27^ ^]^ Specifically, the characteristic peaks of Pd/C at 40°, 47°, and 68° can be indexed to the (111), (200), and (220) planes of cubic Pd (PDF#88‐2335), respectively, implying the successful synthesis of counterpart Pd. Meanwhile, the characteristic peaks of a‐PdGa/C and a‐PdSn/C can also be indexed to the cubic Pd with a negative shift. This fact indicates that the Ga or Sn atom leads to the increase of lattice spacing between Pd atoms, illustrating the successful formation of alloy.^[^ ^28^, ^29^ ^]^ Moreover, the XRD peaks of i‐Pd_2_Ga/C and i‐Pd_2_Sn/C matched well with the orthorhombic Pd_2_Ga (PDF#65‐7607) and Pd_2_Sn (PDF#65‐1293), respectively, confirming the successful synthesis of intermetallic compounds.^[^ ^30^ ^]^ Meanwhile, the diffraction peaks of the Pd/C counterpart aligned well with the cubic Pd (PDF#88‐2335). To further investigate the elemental composition and chemical states, X‐ray photoelectron spectroscopy (XPS) was conducted.^[^ ^31^, ^32^ ^]^ The high‐resolution Pd 3d XPS spectra (Figure 1b) revealed that the binding energies of the metallic Pd 3d_5/2_ peak in a‐PdGa/C, i‐Pd_2_Ga/C, a‐PdSn/C, and i‐Pd_2_Sn/C were located at 335.9, 335.8, 336.1 eV, and 335.8 eV, respectively, showing a notable positive shift compared to Pd/C counterpart.^[^ ^33^ ^]^ This fact indicates that electron transfer from Pd to Ga or Sn among aforementioned catalysts. In other words, the introduction of p‐block Ga or Sn elements could result in a reduction in the electron cloud density around Pd and an increase in its chemical valence state. Concurrently, the peaks for these prepared Pd‐based materials located at ≈337.0 eV can be indexed to the 3d_5/2_ of oxidized Pd, which were caused by the inevitable exposure to air.^[^ ^34^ ^]^ Besides, the Ga 2p XPS spectra revealed the coexistence of metallic Ga and oxidized Ga^x^⁺ in a‐PdGa/C and i‐Pd_2_Ga/C.^[^ ^35^ ^]^ Specifically, the high‐resolution Ga 2p spectra of a‐PdGa/C and i‐Pd_2_Ga/C displayed two sets of peaks at 1116.6 and 1143.6 eV (metallic Ga), and 1118.9 and 1145.7 eV (oxidized Ga^x+^) for the 2p_3/2_ and 2p_1/2_ orbitals, respectively (Figure 1c; Figure S1, Supporting Information). Similarly, the Sn 3d XPS spectra of a‐PdSn/C and i‐Pd_2_Sn/C showed two sets of peaks at 485.7 and 494.1 eV (metallic Sn), and 487.0 and 495.3 eV (oxidized Sn^x+^) for the 3d_5/2_ and 3d_3/2_ orbitals, respectively, confirming the coexistence of metallic and oxidized Sn species.

As characterized by transmission electron microscopy (TEM), it can be observed that all synthesized samples exhibited nanoparticle morphology (Figure 1d; Figure S2, Supporting Information).^[^ ^18^, ^36^, ^37^ ^]^ Subsequently, with the statistical analysis of particle size distribution, the average particle sizes of a‐PdGa/C, i‐Pd_2_Ga/C, a‐PdSn/C, i‐Pd_2_Sn/C, and Pd/C were measured to be ≈6.91, 6.59, 6.89, 6.86, and 6.06 nm, respectively, as depicted in Figure S3 (Supporting Information). Typically, the a‐PdGa/C was further characterized using high‐resolution TEM (HRTEM), intensity profiles, and high‐angle annular dark‐field scanning transmission electron microscopy (HAADF‐STEM) combined with energy‐dispersive X‐ray spectroscopy (EDX).^[^ ^38^, ^39^ ^]^ Specifically, as characterized by the HRTEM, it can be observed that the lattice fringes with a lattice spacing was measured to be 0.223 nm, in line with the Pd (111) plane (Figure 1e,f).^[^ ^40^ ^]^ This result can be further verified by the corresponding filtered inverse fast Fourier transform image (Figure 1g) and intensity profiles of a‐PdGa/C (Figure 1h), validating the success in the introduction of p‐block element and the formation of alloy catalysts. Furthermore, its HAADF‐STEM‐EDX elemental mappings (Figure 1i) manifested the existence and uniform distribution of Pd and Ga throughout the sample.

**Figure 2:**
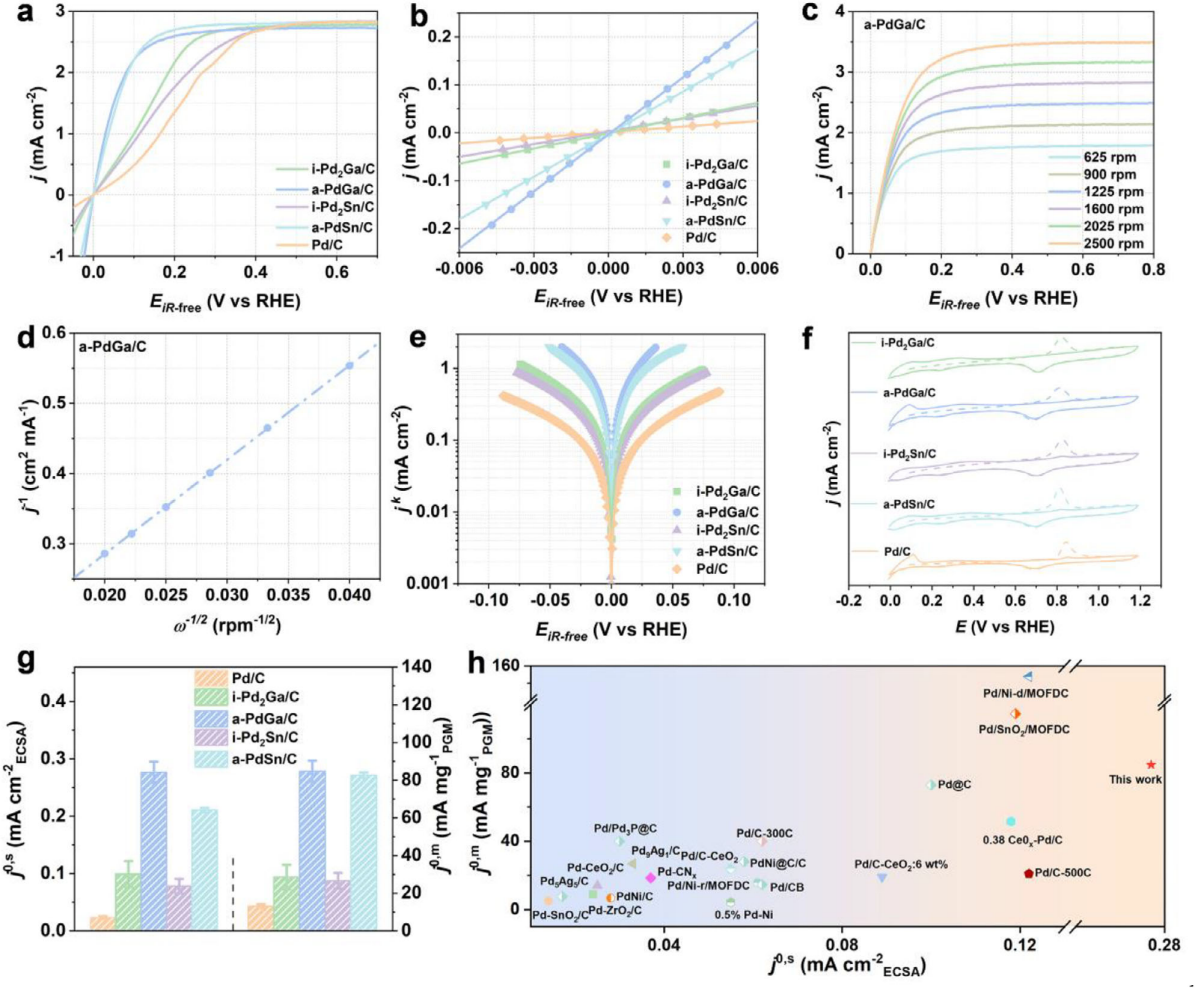
a) HOR polarization plots in H_2_‐saturated 0.1 m KOH at a scan rate of 10 mV s^−1^ with a rotation rate at 1600 rpm, b) Linear‐fitting curves in the micro‐polarization region for a‐PdGa/C, i‐Pd_2_Ga/C, a‐PdSn/C, i‐Pd_2_Sn/C, and Pd/C. c) HOR polarization curves of the a‐PdGa/C at different rotation rates. d) Koutecky–Levich plot obtained from a‐PdGa at an overpotential of 0.6 V. e) Tafel plots with the Butler–Volmer fitting lines for a‐PdGa/C, i‐Pd_2_Ga/C, a‐PdSn/C, i‐Pd_2_Sn/C, and Pd/C. f) The CO stripping curves in Ar‐saturated 0.1 m KOH with a scan speed of 20 mV s^−1^ for a‐PdGa/C, i‐Pd_2_Ga/C, a‐PdSn/C, i‐Pd_2_Sn/C, and Pd/C. g) Exchange current densities normalized by relevant precious metal loadings (*j* ^0,m^) and ECSA (*j* ^0,s^). h) Comparison of the *j* ^0,m^ and *j* ^0,s^ with those of reported Pd‐based catalysts (only consisting of Pd metal among noble metals).

The electrocatalytic behaviors of aforementioned prepared catalysts were evaluated through a rotating disk electrode (RDE) with a typical three‐electrode system (see Experimental Section for details). First, the HOR polarization curves were obtained at a rotation speed of 1600 rpm and a scan rate of 10 mV s^−1^ in a hydrogen‐saturated 0.1 m KOH electrolyte (**Figure** **2**a).^[^ ^41^, ^42^, ^43^ ^]^ The experimental results indicated that a‐PdGa/C exhibited the best apparent activity among the tested catalysts. Notably, the exchange current density (*j* ^0^) of electrocatalysts, the pivotal parameter of intrinsic activity, can be determined from two strategies. One method is to linearly fit the data from their polarization curves in the micro‐polarization region (−5 to 5 mV), according to the approximate Butler–Volmer formula, as illustrated in Figure 2b.^[^ ^44^ ^]^ Concurrently, the other method to calculate the *j* ^0^ is through non‐linear fitting on the basis of Butler–Volmer formula. Specifically, the polarization curves were first carried out at varied rotation speeds ranging from 2500 to 625 rpm (Figure 2c; Figure S4, Supporting Information).^[^ ^45^, ^46^ ^]^ It can be observed that with the increase of rotation speed, the limiting current density is enhanced, indicating that higher rotation speed facilitated the hydrogen mass transport. To exclude the interference of hydrogen mass transfer and attain kinetic current density (*j* ^k^), Koutecky–Levich plots were further constructed for these obtained Pd‐based catalysts (Figure 2d; Figure S5, Supporting Information).^[^ ^47^ ^]^ Besides, the inductively coupled plasma atomic emission spectroscopy (ICP‐AES) was employed to quantify the platinum group metal (PGM) loadings in these catalysts.^[^ ^48^ ^]^ Accordingly, after being normalized by the Pd loading, the *j* ^k,m^ at 50 mV can be calculated to be 222.10, 180.82, 57.94, 52.6, and 25.10 mA mg_PGM_^−1^ for a‐PdGa/C, a‐PdSn/C, i‐Pd_2_Ga/C, i‐Pd_2_Sn/C, and Pd/C, respectively, as listed in Table S1 (Supporting Information). The order of *j* ^k,m^ is a‐PdGa/C > a‐PdSn/C > i‐Pd_2_Ga/C > i‐Pd_2_Sn/C > Pd/C, which initially suggests that the introduction of p‐block Ga or Sn is conducive to enhancing the alkaline HOR kinetics for Pd‐based catalysts. To further obtain the *j* ^0^ through non‐linear fitting method, the relationship between the logarithm of the *j* ^k^ and the potential, along with the corresponding Butler‐Volmer fitting result, was presented in Figure 2e.^[^ ^49^, ^50^, ^51^ ^]^ Additionally, the electrochemical surface area (ECSA) of the catalysts was primarily determined from the carbon monoxide (CO) stripping peaks in the cyclic voltammetry (CV) curves (Figure 2f). For comparison, the ECSA values were also estimated using the PdO reduction peak method (Figure S6, Supporting Information),^[^ ^19^ ^]^ and both approaches yielded consistent trends. On these bases, the *j* ^0^, which is calculated through the above two methods, was further normalized by the ECSA and the PGM (Pd) loading to acquire the specific activities (*j* ^0,s^) and mass activities (*j* ^0,m^) of these prepared catalysts, respectively, as listed in Table S1 (Supporting Information).^[^ ^52^, ^53^ ^]^ It can be determined that the order of *j* ^0,s^ and *j* ^0,m^ for these catalysts, obtained by the above two methods, is consistent. It is noteworthy that a‐PdGa/C exhibited the highest *j* ^0,s^ and *j* ^0,m^ among all samples, reaching 0.277 mA cm^−2^ and 84.77 mA mg_PGM_^−1^, respectively (Figure 2g). More remarkably, the *j* ^0,s^ of a‐PdGa/C is much better than other Pd‐based catalysts in this catalytic system, and even nearly sixfold better than that of Pd/C counterpart (0.024 mA cm^−2^). With regard to the *j* ^0,m^, the a‐PdGa/C still outperformed the a‐PdSn/C (82.71 mA mg_PGM_^−1^), i‐Pd_2_Ga/C (28.60 mA mg_PGM_^−1^), i‐Pd_2_Sn/C (26.62 mA mg_PGM_^−1^), and Pd/C (13.15 mA mg_PGM_^−1^), exhibiting the best *j* ^0,m^. Based on aforementioned results, it can be seen that the alkaline HOR performance of the obtained Pd‐based catalysts with the introduction of either Ga or Sn is superior to that of Pd/C counterpart. The enhanced catalytic performance of these Pd‐based samples can be attributed to the enhancement of electronic interaction, as confirmed by the above XPS results, which might be induced by the d‐p orbital hybridization between p‐block atoms (Ga or Sn) and d‐block atoms (Pd). Regarding the alkaline HOR activity toward decent Pd‐based alloy catalysts, the catalyst introduced with Ga element far exceeds that introduced with Sn element, which will be further explained later in combination with theoretical calculation.

Furthermore, Figure 2h and Table S2 (Supporting Information) provide a comparison of the alkaline HOR performance of these obtained catalysts with that of other reported Pd‐based catalysts (only consisting of Pd metal among noble metals), which further demonstrate that a‐PdGa/C possesses with outstanding catalytic activity in *j* ^0,s^ and *j* ^0,m^, especially, *j* ^0,s^ among Pd‐based catalysts. Apart from the catalytic activity toward HOR catalysts, stability is also a critical parameter for evaluating electrocatalytic performance.^[^ ^22^, ^36^ ^]^ Typically, to investigate the stability of the a‐PdGa/C catalyst, the accelerated durability test (ADT) was conducted with 3000 CV cycles. By comparing the HOR polarization curves and CV curves before and after the ADT, Figure  S7 (Supporting Information) showed almost negligible degradation during the alkaline HOR, indicating its excellent stability (Figure  S7, Supporting Information). Subsequently, the characterization of XRD, TEM, size distribution and XPS was executed for the sample after the durability test, which further revealed that the a‐PdGa/C remained stable without significant changes during ADT (Figures S8–S10, Supporting Information).

**Figure 3:**
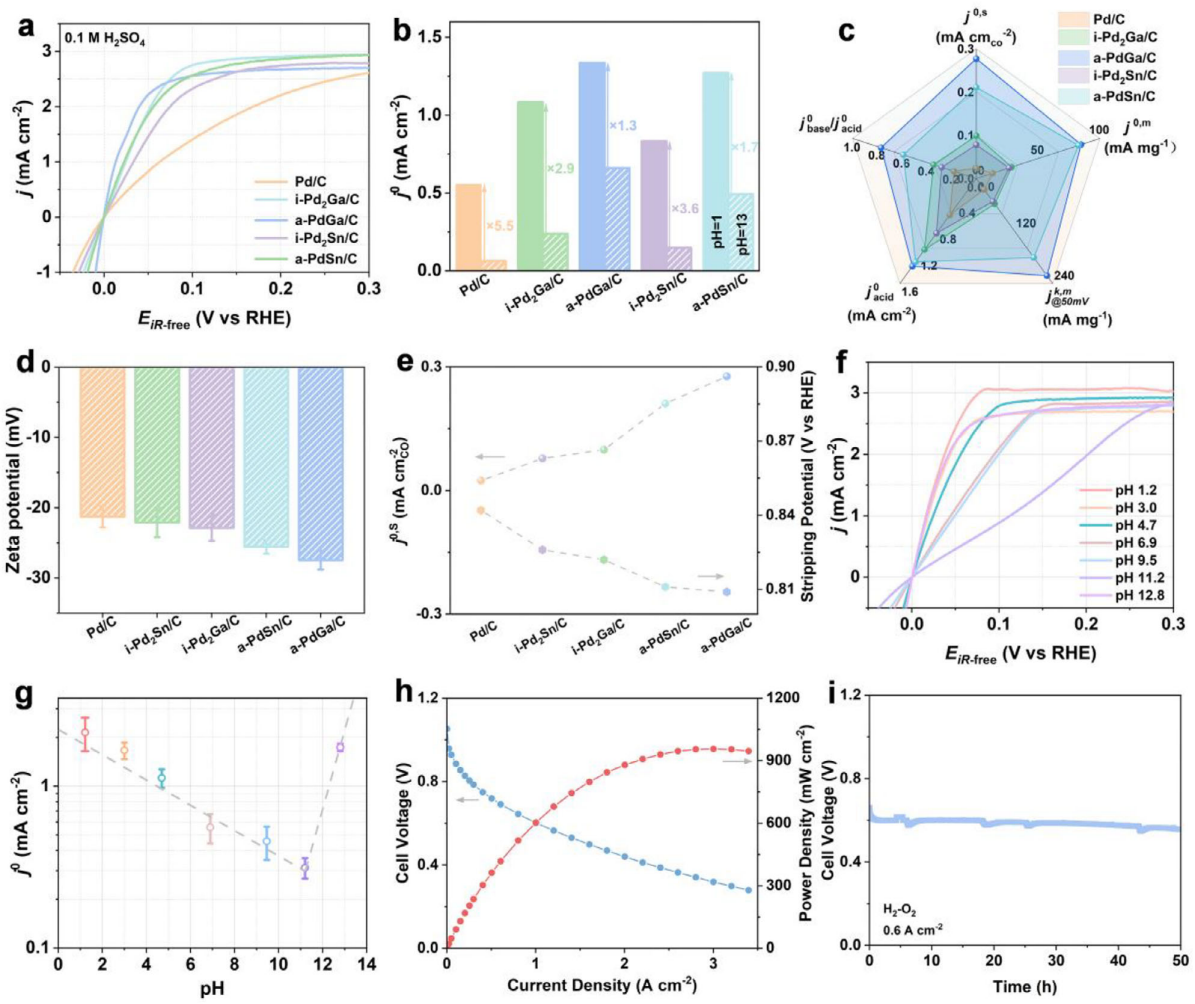
a) HOR polarization curves in H_2_‐saturated 0.1 m H_2_SO_4_ at a rotating speed of 1600 rpm and a scan rate of 10 mV s^−1^, b) Comparison of *j* ^0^ in acid solution (pH 1) and alkaline solution (pH 13), c) Comparison of *j* ^0,s^, *j* ^0,m^, *j* ^k,m^_@50mV_, *j* ^0^_acid_ and *j* ^0^_base_/*j* ^0^_acid_, d) Zeta potential for a‐PdGa/C, i‐Pd_2_Ga/C, a‐PdSn/C, i‐Pd_2_Sn/C and Pd/C. e) Relationship between the CO stripping potential and *j* ^0,s^ of a‐PdGa/C, i‐Pd_2_Ga/C, a‐PdSn/C, i‐Pd_2_Sn/C and Pd/C. f) HOR polarization curves of a‐PdGa/C measured in varied H_2_‐saturated electrolytes ranging from pH 1 to 13 at a rotating speed of 1600 rpm and a scan rate of 10 mV s^−1^. g) Non‐monotonous variation of *j* ^0^ with pH for a‐PdGa/C. h) H_2_–O_2_ AEMFC performance with an a‐PdGa/C anode catalyst and a commercial Pt/C cathode catalyst. The anode and cathode PGM loadings are 0.24 and 0.3 mg cm^−2^. i) Stability test with a a‐PdGa/C anode catalyst and a commercial Pt/C cathode catalyst.

As is well known, a significant decrease in HOR kinetics can be resulted by the electrolytes varying from acid to base. Accordingly, reducing the kinetic gap between acidic and alkaline electrolytes (acid–base kinetic gap) is thus indispensable for developing high‐performance HOR electrocatalysts. To address this challenge, the HOR behaviors of these prepared catalysts were further evaluated under acidic conditions.^[^ ^25^ ^]^ For comparison, polarization curves were recorded for all catalysts in acidic electrolytes (**Figure** **3**a), and the *j* ^0^ were calculated through the linear fitting method (Figure 3b).^[^ ^54^, ^55^ ^]^ Interestingly, the order of *j* ^0^ under acidic environment is consistent with that of under alkaline environment, and the trend of acid–base kinetic gap also aligns with the order of acidic/alkaline activity. More critically, the results revealed that a‐PdGa/C exhibited the smallest acid–base kinetic gap. On these bases, a radar plot was further constructed, as presented in Figure 3c, which further illustrates the comprehensive advantages of a‐PdGa/C in multiple performance evaluation dimensions, including *j* ^k,m^ at 50 mV, *j* ^0,m^, *j* ^0,s^, *j* ^0^ under acidic condition (*j* ^0^_acid_), and the ratio of alkaline *j* ^0^ to acidic *j* ^0^ (*j* ^0^_base_/*j* ^0^_acid_).^[^ ^56^ ^]^ In addition, the characterization of Zeta potential was further conducted, which provides information about the charge capacity of the catalyst and its interaction with OH species. As shown in Figure 3d, the Zeta potential of a‐PdGa/C reached −27.5 mV, which is significantly more negative than other catalysts, including a‐PdSn/C (−25.6 mV), i‐Pd_2_Ga/C (−22.9 mV), i‐Pd_2_Sn/C (−22.1 mV), and Pd/C (−21.3 mV). This pronounced negative charge indicates that a‐PdGa/C exhibits stronger OHBE, thereby promoting the HOR kinetics. The activity trend for these prepared catalysts aligns with the order of OHBE (a‐PdGa/C > a‐PdSn/C > i‐Pd_2_Ga/C > i‐Pd_2_Sn/C > Pd/C), as evidenced by the result of Zeta potential, further validating the critical role of OHBE in accelerating HOR kinetics. The relationship between OHBE and HOR activity was further investigated. As shown in Figure 3e, a clear correlation is observed between the CO stripping peak potential and *j* ^0,s^. Compared with Pd/C (0.842 V), i‐Pd_2_Ga/C (0.822 V), i‐Pd_2_Sn/C (0.826 V), and a‐PdSn/C (0.811 V), a‐PdGa/C exhibits a lower CO stripping peak potential of 0.809 V. This fact indicated that a‐PdGa/C possesses the strongest OHBE among these catalysts, and the strengthened order of OHBE is responsible for the trend of HOR activity. Collectively, the strong OHBE of a‐PdGa/C significantly improved its HOR kinetics in alkaline media, and ultimately narrowed the acid–base kinetic gap.

To further explore the fundamental reasons behind the exceptional performance of the a‐PdGa/C catalyst, its HOR behavior was systematically studied across the entire pH range (≈1–13), as shown in Figure 3f.^[^ ^25^ ^]^ To ensure data accuracy, the K^+^ concentration in all buffer solutions was strictly controlled at the same level (0.1 m K^+^) to eliminate the potential influence of alkali metal cations on catalytic performance (Table S3, Supporting Information). Generally, extensive studies have verified that the HBE is considered as a key descriptor for hydrogen electrocatalysis, and the current density generally exhibits a linear relationship with electrolyte pH. However, as for the a‐PdGa/C catalyst, a distinct deviation was observed, as its *j* ^0^ showed a non‐monotonic dependence on pH, with a clear inflection point around pH 11.2 (Figure 3g). This phenomenon suggests that the traditional 1D interpretation of HBE theory may be insufficient to comprehensively describe the reaction mechanism under varying pH conditions. Instead, it demonstrates that the catalytic performance of a‐PdGa/C is influenced by more intricate factors. As is well known, OHBE plays a decisive role in the adsorption and dissociation processes of hydroxide ions, both of which are essential steps toward HOR under higher‐pH environment. Therefore, the OHBE might emerge as a critical complementary parameter for understanding catalytic performance, particularly in alkaline media, in line with the previous conclusion.

Given the outstanding HOR performance of a‐PdGa/C observed in RDE measurements, its practical applicability was further evaluated in an AEMFC. A high‐performance membrane electrode assembly (MEA) was fabricated via a catalyst‐coated membrane (CCM) method, employing a‐PdGa/C as the anode and commercial Pt/C as the cathode, with PGM loadings of 0.24 and 0.3 mg cm^−2^, respectively. The current‐voltage polarization and corresponding power density curves are shown in Figure 3h. Specifically, the MEA based on a‐PdGa/C achieved an impressive peak power density (PPD) of up to 950 mW cm^−2^ under H_2_–O_2_ conditions. Furthermore, as illustrated in Figure 3i, the a‐PdGa/C‐based MEA exhibited decent operational durability, sustaining stable performance at a current density of 0.6 A cm^−2^ for over 50 h. These results highlight the great potential of a‐PdGa/C as a highly efficient and durable anode catalyst for practical AEMFC applications.

**Figure 4:**
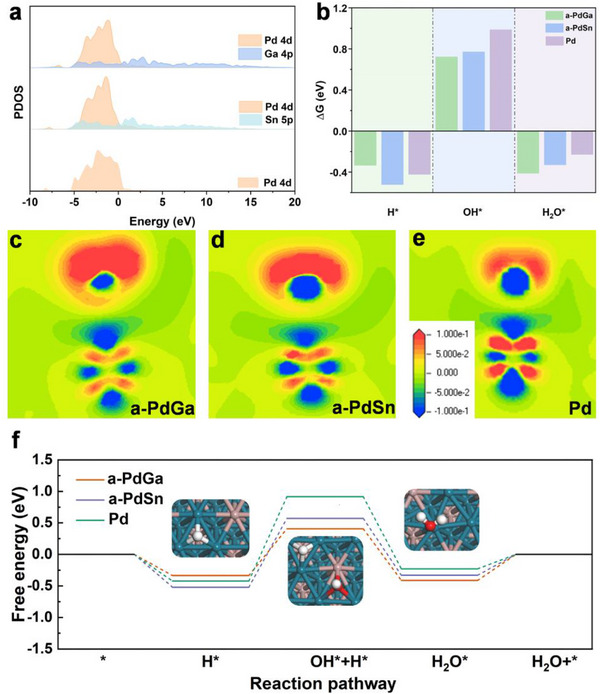
a) The d‐PDOS of Pd and p‐PDOS of Ga in a‐PdGa, the d‐PDOS of Pd and p‐PDOS of Sn in a‐PdSn, as well as the d‐PDOS of Pd. b) Calculated free energies of H^*^, OH^*^, and H_2_O^*^ on a‐PdGa, a‐PdSn, and Pd. The charge density difference between Pd and O in the region for OH^*^ on c) a‐PdGa, d) a‐PdSn, and e) Pd. f) The reaction pathways of a‐PdGa, a‐PdSn, and Pd for alkaline HOR. The insets are theoretical models of a‐PdGa.

To gain deeper insights into the remarkable HOR performance of a‐PdGa/C, density functional theory (DFT) calculations were further performed. Based on the results from XRD and HRTEM, the (111) crystal facets of Pd, a‐PdSn, and a‐PdGa were selected and constructed as the computational models (Figures S11–S13, Supporting Information). As shown in **Figure** **4**a, in terms of the a‐PdGa and a‐PdSn alloy systems, significant hybridization occurs between the Pd 4d orbitals and the Ga 4p or Sn 5p orbitals, which demonstrates the intense d‐p orbital hybridization for this alloy system. This fact can lead to the charge transfer between Pd and Ga/Sn, as evidenced by XPS, which is beneficial for the enhancement in HOR kinetics.^[^ ^33^, ^57^ ^]^ Furthermore, the free energy of different reactive species (H, OH, H_2_O species) on the catalytic surface of a‐PdGa, a‐PdSn, and Pd was analyzed to simulate their adsorption behavior (Figures S14–S16, Supporting Information).^[^ ^58^ ^]^ As shown in Figure 4b, the hydrogen adsorption free energies (Δ*G*_H*_) of a‐PdGa, a‐PdSn, and Pd are −0.335, −0.521, and −0.423 eV, respectively. It can be concluded that the Δ*G*_H*_ of a‐PdGa is the closest to its optimal value (Δ*G*_H*_ = 0), demonstrating that a‐PdGa possesses with the optimal HBE among the three catalysts. Unfortunately, these values (a‐PdGa < Pd < a‐PdSn) are not fully correlate with the trend of their acid or alkaline HOR activity (a‐PdGa > a‐PdSn > Pd). This finding might suggest that HBE might not be the sole determinant factor toward HOR for this catalytic system, consistent with previous results. Consequently, the HOR kinetics might also be influenced by other reactive species such as OH^*^ or H_2_O^*^.^[^ ^13^ ^]^ With regard to hydroxyl adsorption free energy (Δ*G*_OH*_), the value of a‐PdGa (0.722 eV) is lower than that of a‐PdSn (0.771 eV) and Pd (0.987 eV), implying that a‐PdGa with the strongest OHBE among these catalysts, which accounts for its prominent HOR performance under alkaline electrolyte. Meanwhile, the order of OHBE is a‐PdGa > a‐PdSn > Pd, in accordance with above experimental results. More importantly, the observed HOR activity trend (a‐PdGa>a‐PdSn>Pd) aligns with the trend of OHBE (or Δ*G*_OH*_) rather than HBE (or Δ*G*_H*_), suggesting that OHBE serves as a more effective descriptor of catalytic activity toward HOR, especially, under alkaline condition. According to d‐band theory, the position of the d‐band center is strongly correlated with the adsorption strength of reaction intermediates. To further elucidate the reaction mechanism, the projected density of states (PDOS) of the Pd d orbitals (d‐PDOS) at OH^*^ adsorption sites for the three catalysts were computed and analyzed, as presented in Figure S17 (Supporting Information). Compared to Pd, the d‐band center of a‐PdGa and a‐PdSn exhibits a significant upward shift. This phenomenon can be attributed to the d‐p orbital hybridization, thereby endowing the strengthened OHBE. On the other hand, the charge density difference of the Pd─O bond upon OH adsorption on a‐PdGa, a‐PdSn, and Pd were calculated to further explore the adsorption behavior of OH species, as depicted in Figures 4c–e. Compared to the Pd counterpart, a‐PdGa and a‐PdSn exhibit a lower degree of electron localization around oxygen along the Pd─O bond, indicating stronger Pd─O interactions.^[^ ^59^ ^]^ Among them, a‐PdGa, with the introduction of p‐block Ga atoms, shows the largest degree of electron delocalization around oxygen along the Pd─O bond, in agreement with its strongest OHBE. This result further corroborates that the OHBE of the Pd‐based catalysts with introducing p‐block elements can be modulated by the charge transfer, induced by the d‐p orbital hybridization strengthen, thereby promoting the HOR kinetics under alkaline conditions.

Considering that H_2_O species is an essential intermediate during HOR process under either acidic or alkaline conditions, the adsorption behaviors of water were further analyzed, as presented in Figure 4b and Figures S14–S16 (Supporting Information). Remarkably, after d‐p hybridization, the adsorption free energy of H_2_O (Δ*G*_H2O*_) for a‐PdGa (−0.412 eV) and a‐PdSn (−0.330 eV) was stronger than that of Pd (−0.229 eV), responsible for the enhanced acidic or alkaline HOR kinetics after introducing p‐block elements. Accordingly, with the consideration of Δ*G*_H2O*_, HBE_app_ of the three catalysts was further calculated, according to the formula (Δ*G*_H,app_ = Δ*G*_H*_– Δ*G*_H2O*_). As presented in Figure S18 (Supporting Information), the HBE_app_ (Δ*G*_H,app_) of a‐PdGa (Δ*G*_H,app_ = 0.077 eV) was the closest to the optimal value (Δ*G*_H,app_ = 0), whereas the strengthened order of HBE_app_ was in good agreement with the trend of acidic or alkaline activities (a‐PdGa > a‐PdSn > Pd). Consequently, we speculated that the HBE_app_ rather than HBE plays an important role in HOR, especially under acidic condition. Furthermore, the reaction paths for alkaline HOR on a‐PdGa, a‐PdSn, and Pd were further explored (Figure 4f). Since the HBE of all catalysts is much stronger than their OHBE, the first step of the reaction is exothermic H adsorption step. The following step is the OH adsorption step, with the energy of 0.742, 1.092, and 1.339 eV for a‐PdGa, a‐PdSn, and Pd, respectively. The subsequent water formation step is exothermic, whereas the final water desorption step is endothermic. On these bases, it is obvious that the OH adsorption step (from H^*^ to H^*^ + OH^*^) is the potential‐determining step (PDS) for the three catalyst. After d‐p orbital hybridization, the energy barrier of PDS for a‐PdGa and a‐PdSn is significantly lower than that of Pd, attributed to their enhanced OHBE, which further underscores the importance of OHBE in optimizing alkaline HOR process. Generally, as the increase the pH of electrolyte, the catalyst surface becomes more negatively charged at HOR potentials, which makes the alkaline metal cations (AM^+^) more crowded near catalyst surface. Accordingly, the strong AM^+^‐water interaction can destroy the connectivity of hydrogen‐bond networks and cause the interfacial hydrogen‐bond gap. Notably, when OH adsorption strengthens on catalytic surface, the coordination between OH^*^ and K^+^ ions would leave more free water molecules, thus leading to the strengthened hydrogen‐bond network and the accelerated mass transfer process. On these bases, it might be deduced that the strengthened OHBE and the interaction of OH and H_2_O species would facilitate connectivity of hydrogen‐bond networks in electric double layer, thus promoting the HOR performance at higher‐pH environment. Collectively, in response to this catalytic system, we assume that the H and H_2_O species might play dominant role for HOR in lower‐pH electrolytes, whereas the OH and H_2_O species are essential for HOR in higher‐pH electrolytes, accounting for the trend of acidic activity or alkaline activity for these catalysts. Concurrently, it can be inferred that the decisive reaction species are different for HOR under different pH electrolytes, which might be responsible for the former acid–base kinetic gap and inflection‐point behaviors.

## 3 Conclusion

In summary, we employed a colloidal synthesis strategy to construct the d‐p hybridized Pd‐based catalytic system (comprising alloy and intermetallic compounds) through the incorporation of p‐block Ga/Sn elements. Among these catalysts, the a‐PdGa/C catalyst exhibited the highest alkaline HOR activity, with a specific activity of 0.277 mA cm^−2^ and a mass activity of 84.77 mA mg_PGM_^−1^. It is noteworthy that the d‐p orbital hybridization within this catalytic system significantly modulated the electronic structure of the active sites, thus optimizing the reaction intermediate behaviors and facilitating the HOR process. Interestingly, the a‐PdGa/C catalyst also displayed exceptional acidic HOR activity along with the smallest acid–base kinetic gap among these catalysts. Importantly, the trend in acidic activity for this constructed catalytic system is consistent with that of HBE_app_, whereas its trend in alkaline activity is associated with OH and H_2_O species. By integrating the experimental results with theoretical calculations, it can be inferred that the decisive reaction species for HOR vary with increasing pH, namely, it transitions from H/H_2_O species in acidic conditions to OH/H_2_O species in alkaline conditions, which is responsible for the observed trends in alkaline/acidic activity as well as the acid–base kinetic gap. These findings offer valuable insights into comprehending the pH‐dependent electrocatalytic behaviors and exploiting exceptional HOR catalysts, which favor the commercial development of AEMFC.

## Conflict of Interest

The authors declare no conflict of interest.

## Supporting information



Supporting Information

## Data Availability

The data that support the findings of this study are available in the supplementary material of this article.
