# Supplementary material for: Narrowing the Kinetic Gap Between Alkaline and Acidic Hydrogen Oxidation Reactions Through Intermediate Behaviors Regulated on D‐p Hybridized Pd‐Based Catalysts
Source: Adv Sci (Weinh). 2025 Oct 14;12(48):e13616. doi: 10.1002/advs.202513616 (PMC12752547; doi:10.1002/advs.202513616)
Supplement: Supplementary file 1 — Supporting Information [file ADVS-12-e13616-s001.docx]

Supporting Information

**Narrowing the kinetic gap between alkaline and acidic hydrogen oxidation reactions through intermediate behaviors regulated on d-p** **hybridized Pd-based catalysts**

Lixin Su, Hao Wu, Shengnan Zhou, Runjie Qian, Chenxi Cui, Shaokun Zhang, Liqing, Wu, Wenting Li, Huan Pang*

**EXPERIMENTAL SECTION**

**Chemicals and Materials.**

Palladium (II) acetylacetonate (Pd(acac)_2_, Changcheng Chemical, > 99%),Gallium (III) 2,4-pentanedionate (Ga(acac)_3_, Bidepharm, 99.991%), Tin (II) acetate (Sn(Ac)_2_, Chemxyz, 98%), Methylamine hydrochloride (MAHC, Aladdin, 98%), oleylamine (OAm, Aladdin Industrial, 80 ~ 90%), hexane (Sinopharm Chemical Reagent, ≥ 97%), absolute ethanol (Sinopharm Chemical Reagent, ≥ 99.7%), sulfuric acid (H_2_SO_4_, Sinopharm Chemical Reagent, 95.0 ~ 98.0%), potassium hydroxide (KOH, Aladdin Industrial, 99.999% metals basis, except sodium), isopropanol (Sinopharm Chemical Reagent, ≥ 99.7%), Nafion® 117 solution (Sigma-Aldrich, ~ 5% in a mixture of lower aliphatic alcohols and water), and were used directly without any further treatment.

**Synthesis of a-PdGa/C.**

Typically, Pd(acac)_2_ (30.5 mg), Ga(acac)_3_ (18.4 mg), OAm (5 mL) and XC-72 carbon (30.0 mg) were added to a two-neck flask and stirred magnetically. Subsequently, the solution was heated to 100 °C and kept for 30 min under vacuum to remove the moisture and oxygen in the reaction system. After the solution was refilled with nitrogen, the temperature was further raised to 220 °C and maintained at this temperature for 60 min. After the resulting solution was naturally cooled to room temperature, the a-PdGa/C samples were centrifuged by adding ethanol and n-hexane and dried at room temperature under vacuum. Finally, the dried samples were put in a quartz boat in the center of a tube furnace and annealed under a gas atmosphere (5% H_2_, 95% N_2_) at 400 °C for 60 min. The temperature of tube furnace was raised at a rate of 5 °C min^−1^.

**Synthesis of a-PdSn/C.**

The synthesis of a-PdSn/C was similar to the a-PdGa/C, except for a fact that the Ga(acac)_3_ (18.4 mg) was changed to Sn(Ac)_2_ (12.0 mg).

**Synthesis of i-Pd_2_Ga/C and i-Pd_2_Sn/C.**

The synthesis of i-Pd_2_Ga/C and i-Pd_2_Sn/C was similar to that of a-PdGa/C and a-PdSn/C, respectively, except for the addition of MAHC (33.8 mg) in the initial synthesis process.

**Synthesis of Pd/C.**

The synthesis of Pd/C was similar to that of a-PdGa/C without the introduction of Ga source.

**Materials characterization.**

The X-ray diffraction (XRD) patterns was performed by Bruker AXS D8 advance with Cu Kα radiation of 40 kV (λ=1.5418 Å). Low-magnification transmission electron microscope (TEM) was conducted on a HITACHI HT7800 at an accelerating voltage of 100 kV. X-ray photoelectron spectra (XPS) were collected with a Thermo Scientific ESCALAB 250Xi X-ray photoelectron spectrometer. High-resolution TEM (HRTEM) images and energy dispersive X-ray spectrometry (EDS) elemental mapping were captured on a Tecnai G2 F30 S-TWIN at an acceleration voltage of 300 kV. Inductively coupled plasma emission spectroscopy (ICP) was conducted on Optima 7300 DV spectrometer.

**Preparation of working electrodes.**

For HOR measurement, 4.0 mg of Pd/C, a-PdGa/C, i-Pd_2_Ga/C, a-PdSn/C or i-Pd_2_Sn/C, was mixed with 2.0 mL of isopropanol solvent containing 0.05 wt% Nafion and ultrasonicated for 30 min to form a homogeneous ink. The glassy carbon (GC) electrode with a diameter of 5 mm was polished with 0.05 mm gamma alumina powder slurry and rinsed with water and ethanol to obtain a neat surface. When the GC electrode was dried under air naturally, 5.0 μL ink was drop-casted on the surface of the GC electrode and dried in air before any electrochemical measurements.

**Electrochemical measurements.**

Electrochemical tests were conducted at CHI 760E electrochemistry workstation. The GC electrode decorated by electrocatalyst served as the working electrode, the graphite rod served as the counter electrode as well as the saturated calomel electrode (SCE) was used as the reference electrode. In this study, all operation was performed at the constant temperature of 303 (± 0.1) K, and all the measured potentials were referred to the reversible hydrogen electrode (RHE) potential with *iR*-compensation. For HOR evaluation, the catalysts were pre-treated under Ar-saturated electrolytes by cyclic voltammetry (CV) curves to obtain a stable voltammogram patterns of Pd/C, a-PdGa/C, i-Pd_2_Ga/C, a-PdSn/C and i-Pd_2_Sn/C samples in the range of -0.02 to 1.22 V. After the fresh electrolyte was H_2_-saturated, the polarization curve was performed at a scan rate of 10 mV s^−1^ under the RDE rotation rate of 1600 rpm in the potential range from about -0.08 to 0.92 V for the Pd/C, a-PdGa/C, i-Pd_2_Ga/C, a-PdSn/C and i-Pd_2_Sn/C samples. The accelerated durability test (ADT) for a-PdGa/C was operated by 3000 CV cycles under N_2_-saturated solution. The precious metal loading on the GC electrode was 10 μg.

Electrochemically active surface area (ECSA) was determined by using CO stripping voltammograms. Typically, CO stripping was conducted by keeping the electrode potential at 0.1 V for 10 min in 0.1 M KOH solution with 99.99% CO for CO adsorbed on metal surface, followed by Ar purging for another 30 min to remove residual CO in the electrolyte. The CO stripping current was obtained via CV in a potential region from 0 to 1.2 V at a sweep rate of 20 mV s^-1^. The value of ECSA (cm^2^) can be calculated by Eq. S1,

$\mathrm{ECSA}_{\mathrm{CO}}= \frac{Q_{\mathrm{CO}}}{Q_{s}}$ Eq. S1

Where Q_CO_ is the measured integral charge, Q_S_ is the surface charge density of 420 μC cm_metal_^-2^which is assumed for a monolayer adsorption of CO on metal.

The estimation of ECSA can be also based on PdO reduction by cyclic voltammogram (CV) recorded in the range of 0.02 to 1.22 V at the sweep rate of 50 mV s^−1^. The value of ECSA (cm^2^) can be calculated by Eq. S2,

$\mathrm{ECSA}_{PdO}= \frac{Q_{\mathrm{PdO}}}{Q_{s}}$ Eq. S2

Where *Q*_PdO_ is the measured integral charge, *Q*_s_ is the surface charge density of 424 μC cm_metal_^−2^.

Electrochemical impedance spectra (EIS) tests were conducted with the AC impedance spectra from 200 kHz to 0.1 kHz and a voltage perturbation of 10 mV. The real part of the resistance at 1 kHz was taken as the uncompensated resistance (*R*_u_) and was used to obtain the *iR*-free potential (*E_iR_*_­free_) according to the following equation (Eq. S3),

$E_{iR­free}=E-iR_{u}$ Eq. S3

where *E* is the measured potential and *i* is the corresponding current.

Kinetic current density (*j^k^*) could be deduced from the Koutecky-Levich equation (Eq. S4),

$\frac{1}{j}=\frac{1}{j^{k}}+\frac{1}{j^{d}}=\frac{1}{j^{k}}+\frac{1}{Bc_{0}\omega^{1/2}}$ Eq. S4

where *j*, *B*, *c*_0_, and *ω* are the measured current density, the Levich constant, the solubility of H_2_ (7.33 × 10^−4^ mol L^−1^), and the speed of the rotating, respectively.

Exchange current density (*j*^0^) could be extracted from the Butler-Volmer equation (Eq. S5),

$j^{k}=j^{0}\left[ e^{\frac{\alpha F}{RT}\eta}-e^{\frac{-\left( 1-\alpha\right)F}{RT}\eta} \right]$ Eq. S5

*j*^0^ could be also obtained from the approximate Butler-Volmer equation (Eq. S6),

$j=j^{0}\frac{\eta F}{RT}$ Eq. S6

where *α*, *η, R*, *T,* and *F* represent the transfer coefficient, the overpotential, the universal gas constant (8.314 J mol^−1^ K^−1^), the temperature in Kelvin (303 K), and the Faraday constant, respectively.

**Membrane-electrode assembly and single-cell test**

The catalyst-coated membrane (CCM, 4 cm^2^) was prepared using Alkymer® W-25 (EVE Hydrogen Enegry Co) as the membrane (25 μm in thickness) with anode side sprayed with the a-PdGa/C catalyst and the cathode side sprayed with the commercial Pt/C catalyst (Johnson–Matthey). The catalyst was also mixed with QAPPT ionomer solution in n-propanol. The catalyst loading was 0.24 mg_PGM_ cm^-2^ for a-PdGa/C and 0.3 mg_PGM_ cm^-2^ for Pt/C. The obtained CCM was immersed in 1.0 M KOH solution overnight to replace Cl^-^ in the membrane to OH^-^. After that, the CCM was washed with ultrapure water for several to remove the excess KOH. The fuel cells were operated on the 850e Multi Range Fuel Cell Test Station (Scribner Associates Co.) at 80 °C. Humidified H_2_ and O_2_ gases were used as the reactant gas with a flow rate at 1000 mL min^-1^ under 0.2 MPa backpressures.

**DFT Calculations**

Density functional theory (DFT) with the generalized gradient approximation (GGA) and Perdew-Burke-Ernzerhof (PBE) exchange-correlation functional was carried out for electronic structure calculations.^[1]^ Ultrasoft pseudopotentials were employed to describe the ionic cores and a plane-wave cutoff of 400 eV was applied to expand the valence electrons, and a Monkhorst-Pack k-point 4× 4 × 1 was used for geometry optimization.^[2]^ The atomic structures were relaxed until the energy convergence criteria 1x10^-5^ eV and force convergence criteria 0.01 eV/Å were reached. For metallic Pd, a four-layers model of the (111) facet was constructed and a 2x2 supercell with a vacuum layer of 15 Å were selected. The topmost layer of the Pd slab model was relaxed while the bottom two layers were fixed. Concurrently, as for the a-PdSn and a-PdSn alloy system, a four-layers model of the (111) facet of Pd replaced Ga or Sn were constructed and a 2x2 supercell with a vacuum layer of 15 Å were selected, respectively. Similarly, the topmost layer of the a-PdSn or a-PdSn slab model was relaxed while the bottom two layers were fixed. The adsorption-free energies of H^*^ (ΔG_H*_), OH^*^ (ΔG_OH*_) and H_2_O^*^ (ΔG_H2O*_) were calculated by ΔG = Δ*E* + Δ*ZPE* - *TΔS*, where Δ*E* is the DFT-based adsorption energy of H, OH or H_2_O, and Δ*ZPE* and *TΔS* are the correction of zero point energy and entropy, respectively.^[3]^


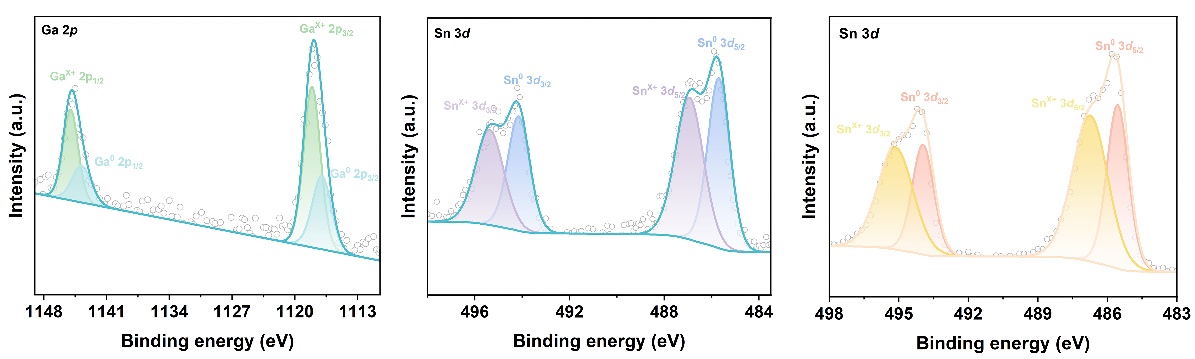


**Figure S1.** The high-resolution XPS of Ga 2p and Sn 3d in i-Pd_2_Ga/C, a-PdSn/C, and i-Pd_2_Sn/C.


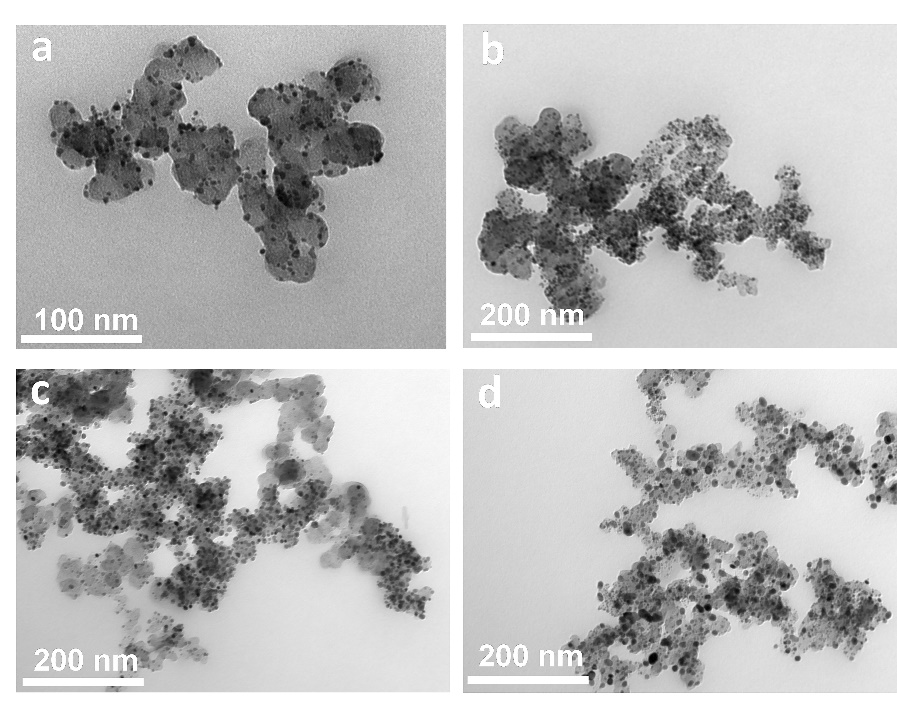


**Figure S2.** TEM images of (a) Pd/C, (b) i-Pd_2_Ga/C, (c) i-Pd_2_Sn/C, and (d) a-PdSn/C.


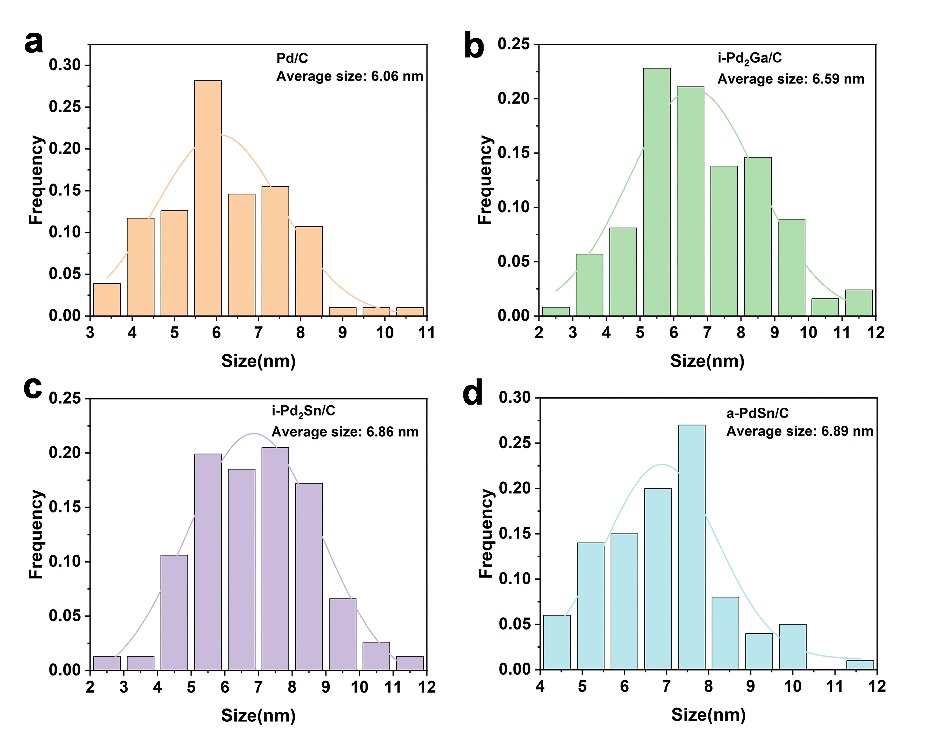


**Figure S3.** Size distribution histograms of (a) Pd/C, (b) i-Pd_2_Ga/C, (c) i-Pd_2_Sn/C, and (d) a-PdSn/C.


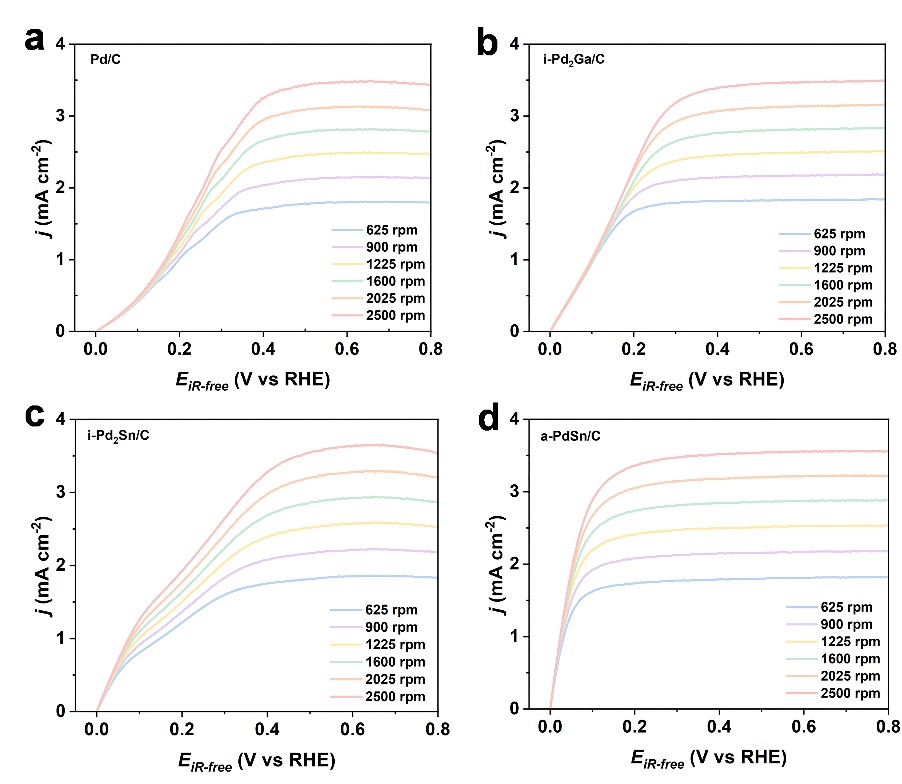


**Figure S4.** HOR polarization curves in H_2_-saturated 0.1 M KOH with a scanning rate of 10 mV s^-1^ at the rotating rates ranging from 2500 to 625 rpm of (a) Pd/C, (b) i-Pd_2_Ga/C, (c) i-Pd_2_Sn/C, and (d) a-PdSn/C.


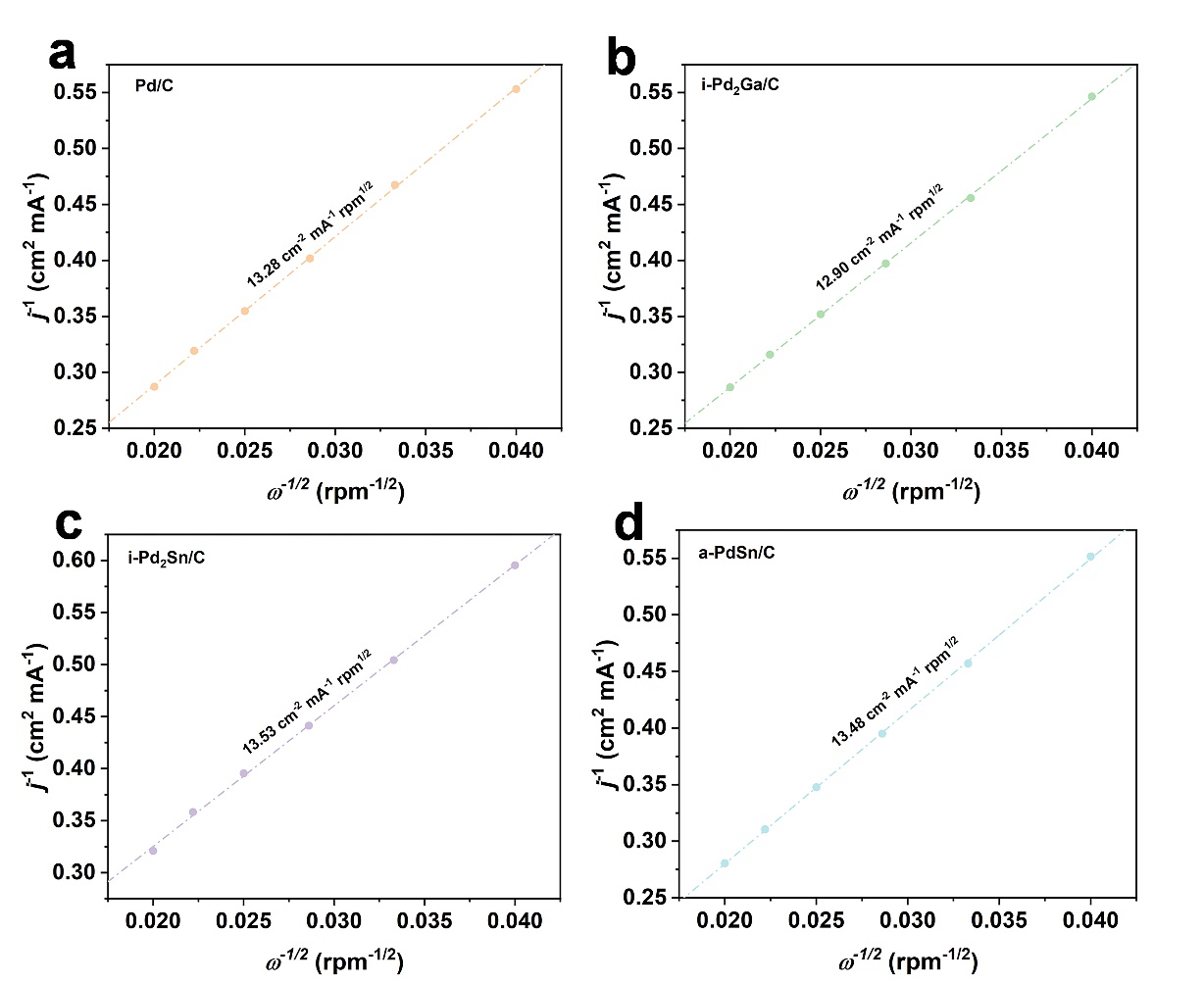


**Figure S5.** Koutecky-Levich plots towards (a) Pd/C, (b) i-Pd_2_Ga/C, (c) i-Pd_2_Sn/C, and (d) a-PdSn/C.

**
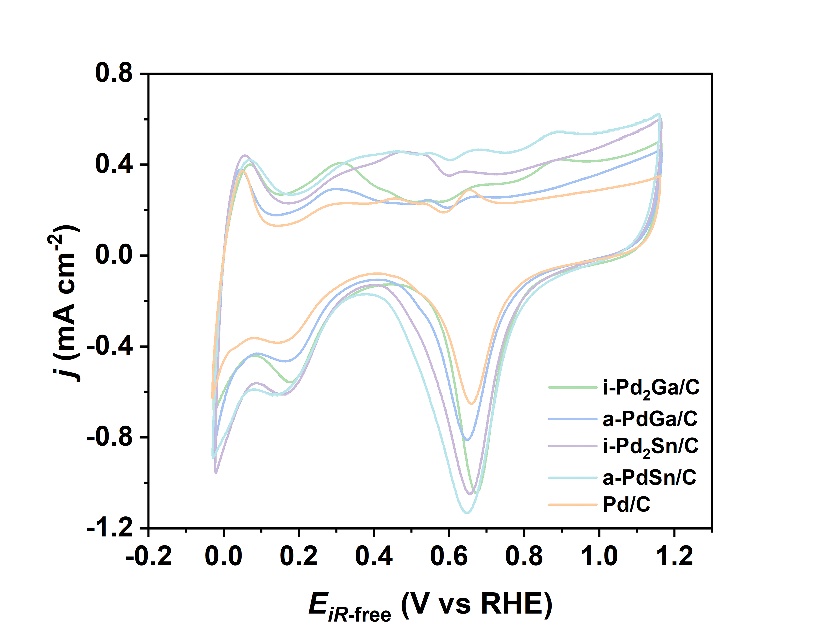
**

**Figure S6.** The CV of i-Pd_2_Ga/C, a-PdGa/C, i-Pd_2_Sn/C, a-PdSn/C and Pd/C.


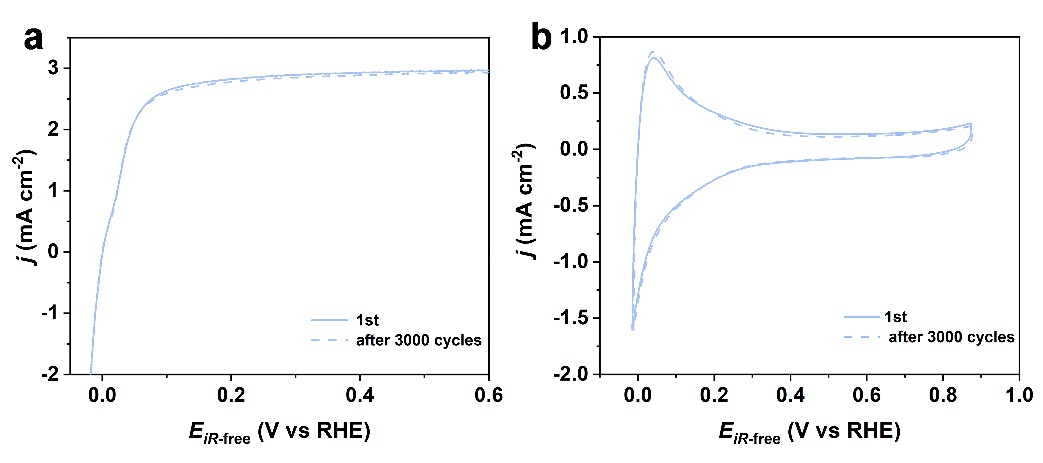


**Figure S7.** (a) HOR polarization curves, (b) CV curves of a-PdGa/C recorded before and after 3000 CV cycles in 0.1 M KOH.


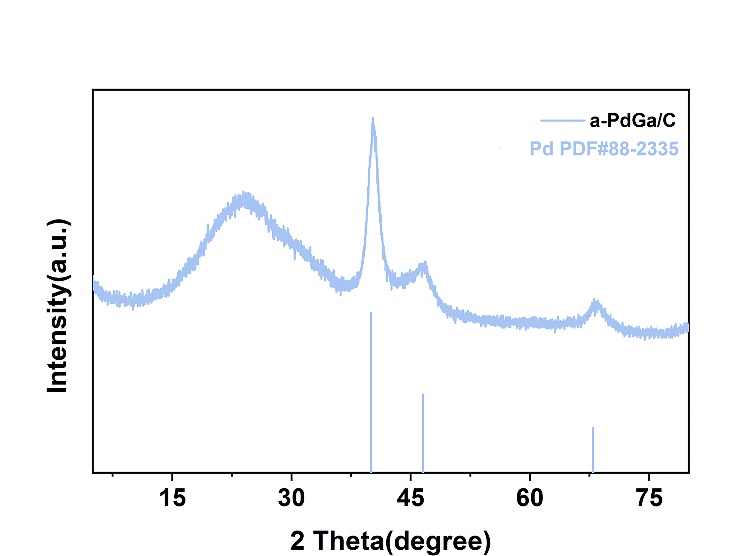


**Figure S8.** XRD pattern of a-PdGa/C recorded after 3000 CV cycles in 0.1 M KOH.


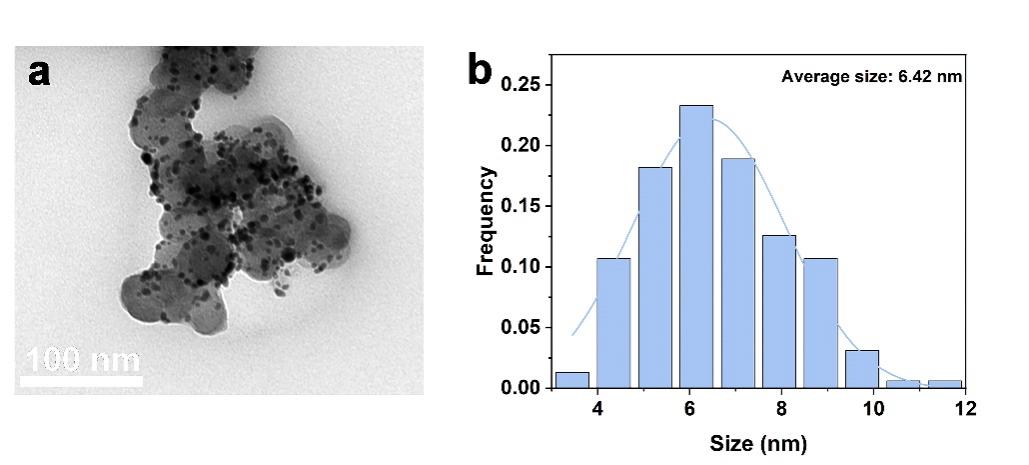


**Figure S9.** (a) TEM image of a-PdGa/C recorded after 3000 CV cycles in 0.1 M KOH; (b) Size distribution histogram of a-PdGa/C recorded after 3000 CV cycles in 0.1 M KOH, derived by counting more than 100 nanoparticles


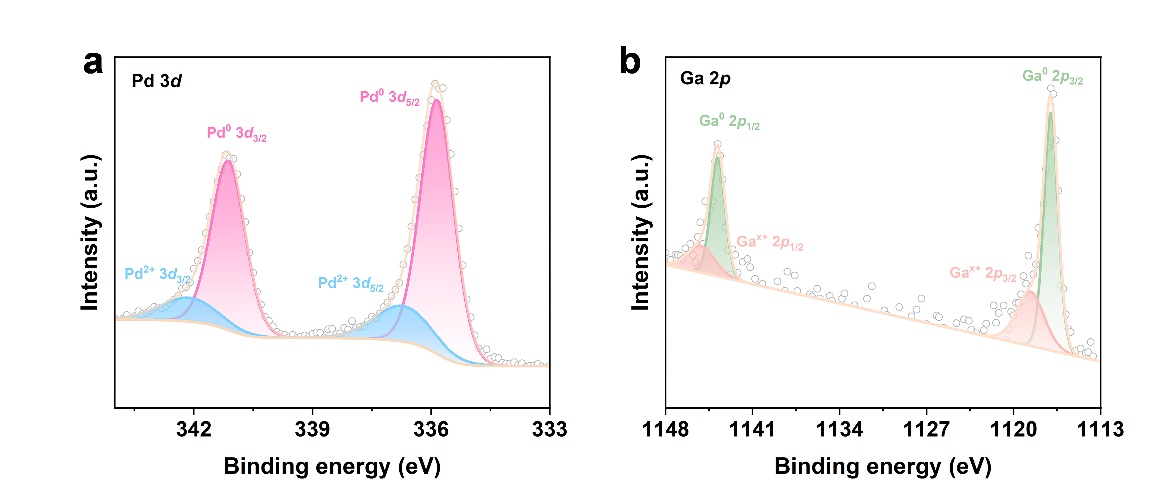


**Figure S10.** High-resolution XPS of (a) Pd 3d, (b) Ga 2p in a-PdGa/C recorded after 3000 CV cycles in 0.1 M KOH.


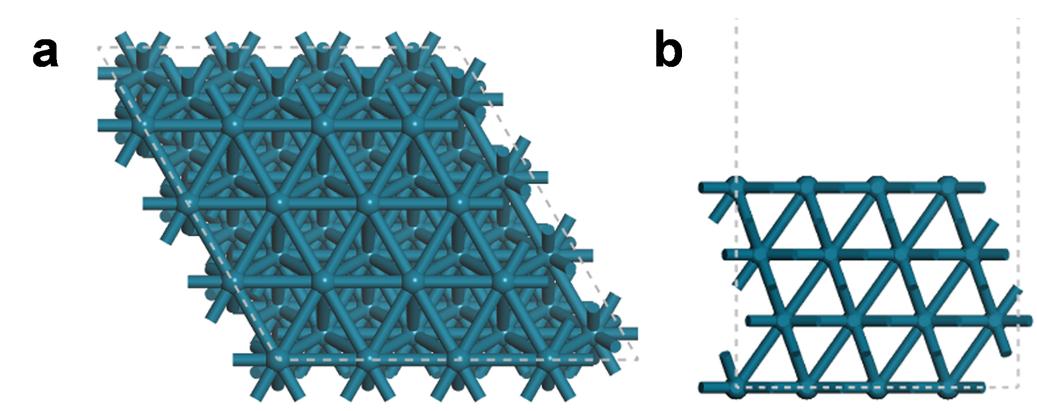


**Figure S11**. (a) The top view of the calculated model for Pd (111) surface. (b) The front view of the calculated Pd (111) model.


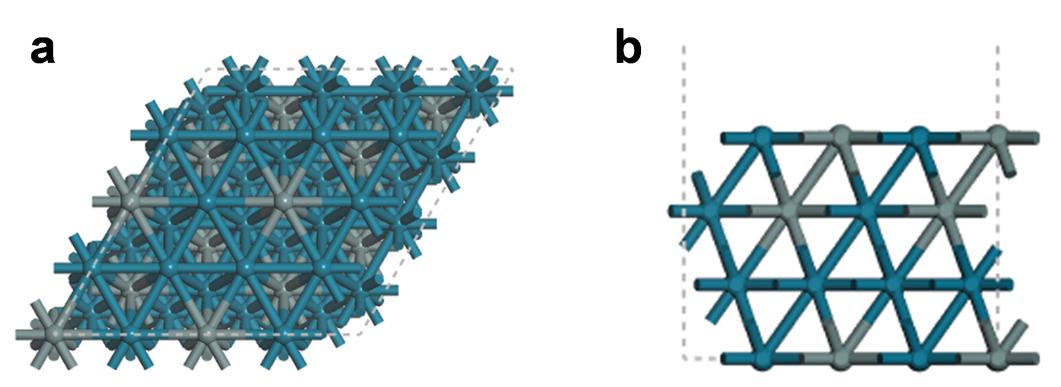


**Figure S12**. (a) The top view of the calculated model for a-PdSn surface. (b) The front view of the calculated a-PdSn model.


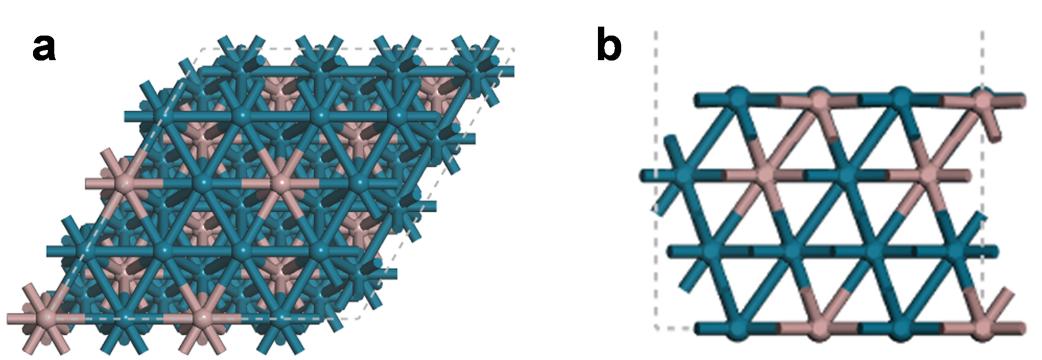


**Figure S13**. (a) The top view of the calculated model for a-PdGa surface. (b) The front view of the calculated a-PdGa model.


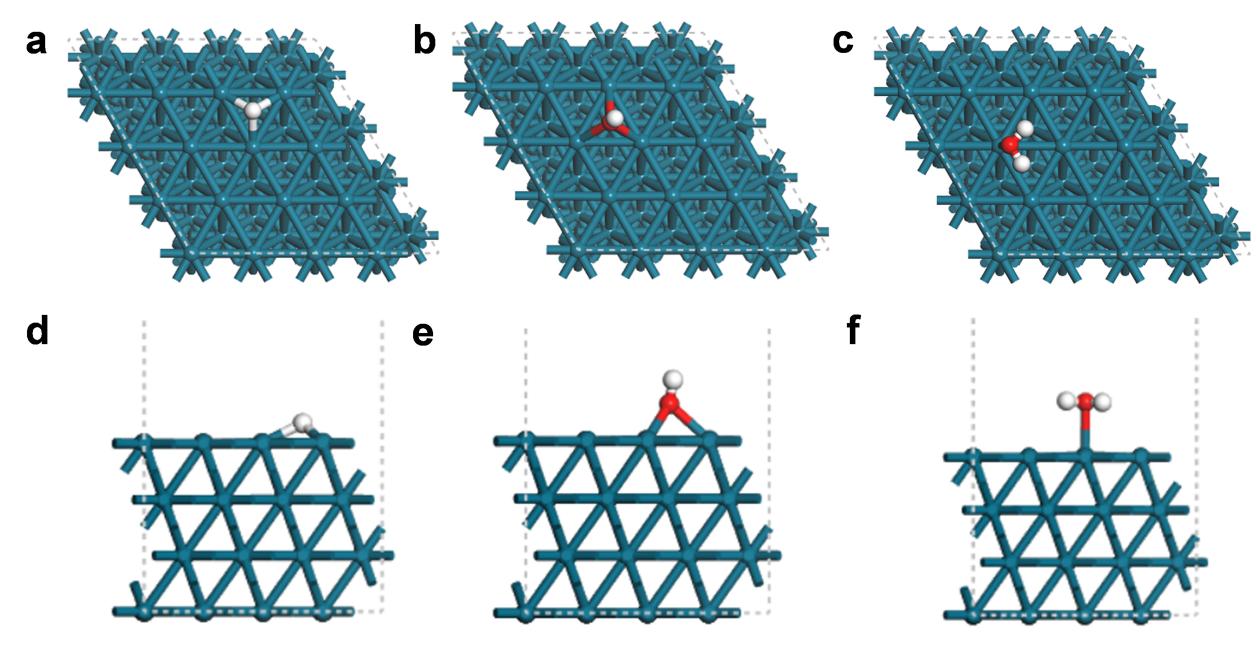


**Figure S14**. The top view of the optimal theoretical structures of intermediates in the process of HOR on Pd (a) H*, (b) OH* and (c) H_2_O*. The front view of the optimal theoretical structures of intermediates in the process of HOR on Pd (d) H*, (e) OH* and (f) H_2_O*.


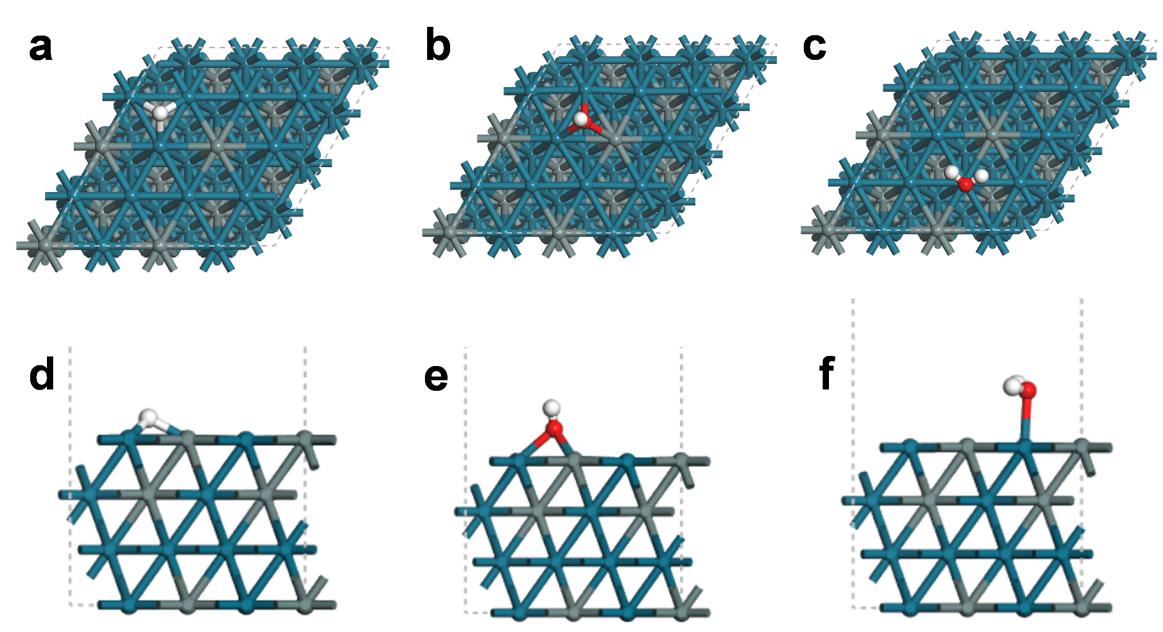


**Figure S15**. The top view of the optimal theoretical structures of intermediates in the process of HOR on a-PdSn (a) H*, (b) OH* and (c) H_2_O*. The front view of the optimal theoretical structures of intermediates in the process of HOR on a-PdSn (d) H*, (e) OH* and (f) H_2_O*.


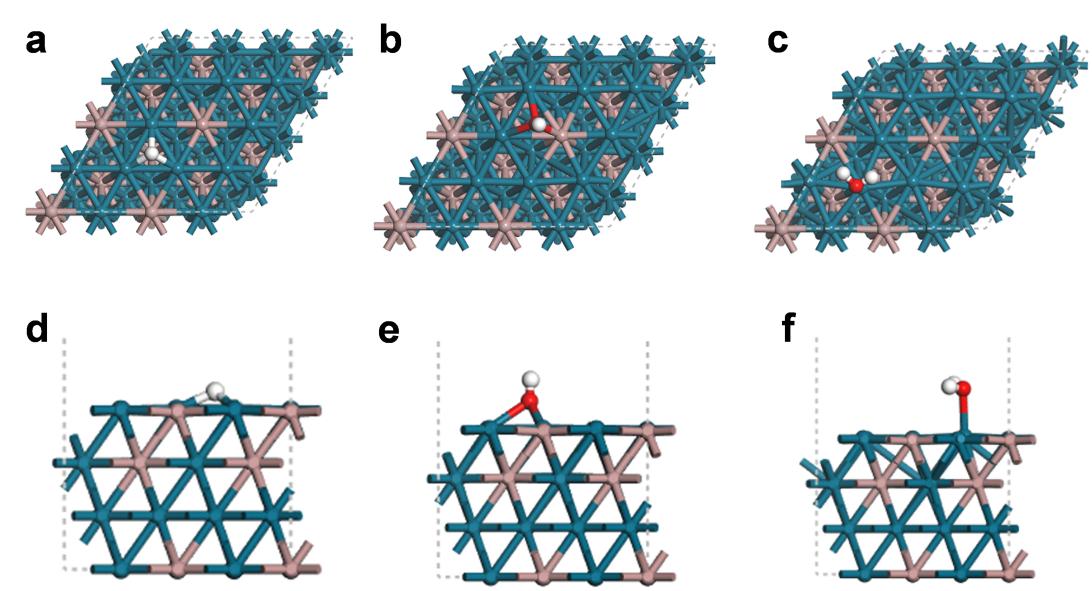


**Figure S16**. The top view of the optimal theoretical structures of intermediates in the process of HOR on a-PdGa (a) H*, (b) OH* and (c) H_2_O*. The front view of the optimal theoretical structures of intermediates in the process of HOR on a-PdGa (d) H*, (e) OH* and (f) H_2_O*.


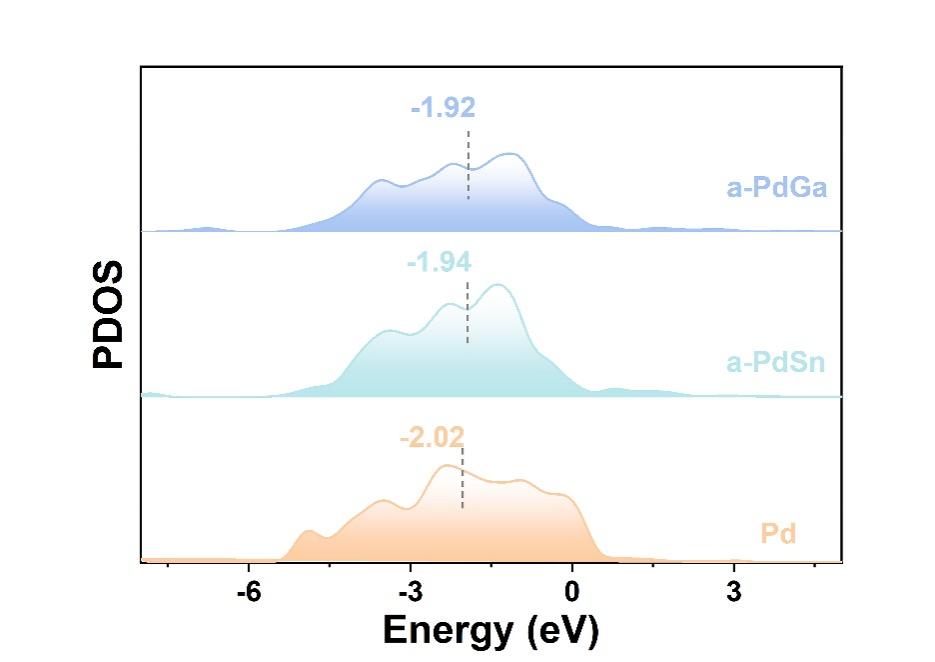


**Figure S17**. The d-orbital projected density of states (d-PDOS) of Pd atoms in the OH* adsorption sites.


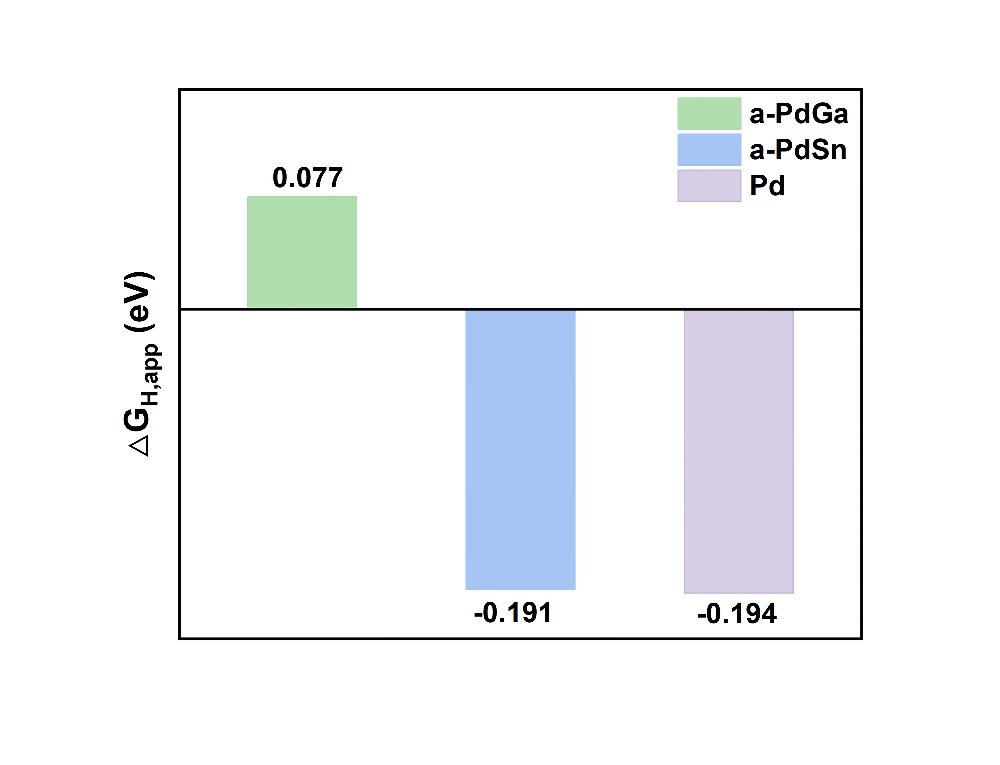


**Figure S18**. The apparent hydrogen adsorption free energy for a-PdGa, a-PdSn and Pd.

**Table S1.** The alkaline HOR activities of catalysts in this work.

| **Sample** | **Butler-Volmer fitting** | | | | **Micropolarization** | | |
| --- | --- | --- | --- | --- | --- | --- | --- |
|  | ***j*^0,s^**  **(mA cm_CO_^−2^)** | ***j*^0,m^**  **(mA mg_PGM_^−1^)** | | ***j*^k,m^_@50mV_**  **(****mA mg_PGM_^−1^)** | ***j*^0,s^**  **(mA cm_CO_^−2^)** | | ***j*^0,m^**  **(mA mg_PGM_^−1^)** |
| Pd/C | 0.024(±0.002) | 13.15(±0.99) | 25.1(±2.404) | | 0.020(±0.002) | 11.43(±1.174) | |
| i-Pd_2_Ga/C | 0.099(±0.023) | 28.60(±6.562) | 57.94(±14.583) | | 0.091(±0.019) | 26.35(±5.586) | |
| **a-PdGa/C** | **0.277(±0.018)** | **84.77(±5.607)** | **222.10(±18.058)** | | **0.255(±0.013)** | **78.15(±4.031)** | |
| i-Pd_2_Sn/C | 0.078(±0.012) | 26.62(±4.221) | 52.67(±9.178) | | 0.070(±0.01) | 23.85(±3.323) | |
| a-PdSn/C | 0.211(±0.004) | 82.71(±1.478) | 180.82(±42.03) | | 0.174(±0.003) | 68.23(±1.372) | |

**Table S2.** Comparison of the HOR activity in 0.1 M KOH referring to pure Pd-based catalysts (only consisting of Pd among noble metals).

| **Catalysts** | ***j*^0,s^ (mA cm_ECSA_^−2^)** | ***j*^0,m^ (mA mg_PGM_^−1^)** | **References** | |
| --- | --- | --- | --- | --- |
| **a-PdGa/C** | **0.277** | **84.77** | This work |  |
| Pd/C | 0.03 | 13.15 |  |  |
| i-Pd_2_Ga/C | 0.093 | 28.6 |  |  |
| i-Pd_2_Sn/C | 0.056 | 26.62 |  |  |
| a-PdSn/C | 0.136 | 82.71 |  |  |
| 0.38 CeO_x_-Pd/C | 0.118 | 51.54 | [4] |  |
| Pd/C | 0.045 | 20.84 |  |  |
| Pd/C-CeO_2_ | 0.055 | 24 | [5] |  |
| Pd/C | 0.0027 | 1.1 |  |  |
| Pd-CNx | 0.037(*j*^0^) | 18.5 | [6] |  |
| Pd/C | 0.005 | / | [7] |  |
| Pd_3_Cu/C | 0.053 | / |  |  |
| Pd_3_Fe/C | 0.073 | / |  |  |
| Pd_3_Co/C | 0.074 | / |  |  |
| Pd-CeO_2_/C | / | 46.4 | [8] |  |
| Pd-CeO_2_(25nm)/C | / | 32.6 |  |  |
| Pd-CeO_2_(5nm)/C | / | 17.8 |  |  |
| Pd/C | / | 14.5 |  |  |
| Pd/C-CeO_2_:  6 wt% | 0.089 | 19 | [9] |  |
| Pd/C-CeO_2_:  10 wt%Pd | 0.055 | 24 |  |  |
| Pd/C-CeO_2_:  20 wt% Pd | 0.083 | 11 |  |  |
| Pd/C | 0.052 | 38 | [10] |  |
| Pd/C-300C | 0.062 | 40 |  |  |
| Pd/C-400C | 0.101 | 34 |  |  |
| Pd/C-500C | 0.122 | 21 |  |  |
| Pd/C-600C | 0.118 | 5 |  |  |
| Pd_9_Ag_1_/C | 0.033 | 26.85 | [11] |  |
| Pd_5_Ag_5_/C | 0.0171 | 7.65 |  |  |
| Pd/C | 0.0352 | 7.93 |  |  |
| Pd-CeO_2_/C | 0.025 | 14 | [12] |  |
| Pd-ZrO_2_/C | 0.024 | 9 |  |  |
| Pd-SnO_2_/C | 0.014 | 5 |  |  |
| Pd/SnO_2_/MOFDC | 0.119 | 114.70 | [13] |  |
| Pd/C | 0.145 | / | [14] |  |
| Pd/CeO_2_/C | 0.214 | / |  |  |
| Pd/CeO_2_/C(mix) | 0.188 | / |  |  |
| PdCu/C-200℃ | 0.016 | 11.29 | [15] |  |
| PdCu/C-500℃ | 0.216 | 127.62 |  |  |
| Pd/C | 0.061 | 37.57 |  |  |
| Pd/Ni-r/MOFDC | 0.061 | 15.67 | [16] |  |
| Pd/Ni-d/MOFDC | 0.122 | 153.88 |  |  |
| Pd/CB | 0.062 | 14.62 |  |  |
| Pd-B_0.1_ | 0.084 | / | [17] |  |
| Pd-B_40_ | 0.077 | / |  |  |
| Pd-B_40sc_ | 0.049 | / |  |  |
| Pd_power_ | 0.08 | / |  |  |
| Pd_SA_/N,S-OPC | 2.019 | / | [18] |  |
| Pd_SA_/N-OPC | 0.861 | / |  |  |
| Pd_SA_/PC | 0.13 | / |  |  |
| Pd-WC_x_ | 1.81 | / | [19] |  |
| V_p_-Pd_3_P@C | 0.85 | 1660 | [20] |  |
| Pd/Pd_3_P@C | 0.03 | 40 |  |  |
| Pd_3.20_P_12_@C | 0.26 | 67 |  |  |
| Pd_3_P@C | 0.35 | 45 |  |  |
| Pd_5_P_2_@C | 0.13 | 18 |  |  |
| Pd@C | 0.1 | 73 |  |  |
| Pd/C-150 | 0.09 | / | [21] |  |
| Pd/C-300 | 0.18 | / |  |  |
| Pd/C-400 | 0.34 | / |  |  |
| Pd/C-500 | 0.29 | / |  |  |
| Pd/C-600 | 0.34 | / |  |  |
| Pd/C-700 | 0.21 | / |  |  |
| 0.1% Pd-Ni | 0.021 | 1.975 | [22] |  |
| 0.5% Pd-Ni | 0.055 | 4.275 |  |  |
| 1% Pd-Ni | 0.016 | 1.35 |  |  |
| Pd@C/C | 0.278 | 24.6 | [23] |  |
| Pd/C | 0.016 | 19.1 |  |  |
| PdNi@C/C | 0.058 | 28.1 |  |  |
| PdNi/C | 0.028 | 6.6 |  |  |

**Table S3.** The preparation of various electrolytes.

| Electrolytes | Acid | | Base | | Total volume  (mL) | pH |
| --- | --- | --- | --- | --- | --- | --- |
|  | **Reagent** | **The amount of substance (mmol)** | **Reagent** | **The amount of substance (mmol)** |  |  |
| H_2_SO_4_ | H_2_SO_4_ | 50 | / | / | 500 | ~1.2 |
| H_3_Ci/KH_2_Ci | H_3_Ci | 100 | KOH | 50 |  | ~3.0 |
| HAc/KAc | HAc | 50 | KAc | 50 |  | ~4.7 |
| KH_2_PO_4_/K_2_HPO_4_ | KH_2_PO_4_ | 16.67 | K_2_HPO_4_ | 16.67 |  | ~6.9 |
| H_3_BO_3_/KB(OH)_4_ | / | / | K_2_B_4_O_7_ | 25 |  | ~9.5 |
| K_2_HPO_4_/K_3_PO_4_ | K_2_HPO_4_ | 10 | K_3_PO_4_ | 10 |  | ~11.2 |
| KOH | / | / | KOH | 50 |  | ~12.8 |

**Reference**

[1] J. P. Perdew, K. Burke, M. Ernzerhof, *Phys. Rev. Lett.* **1996**, *77*, 3865.

[2] D. Vanderbilt, *Phys Rev B*, **1990**, *41,* 7892.

[3] J. K. Nørskov, T. Bligaard, A. Logadottir, J. R. Kitchin, J. G. Chen, S. Pandelov, U. Stimming, *J. Electrochem. Soc.* **2005**, *152,* J23.

[4] R. K. Singh, E. S. Davydova, J. Douglin, A. O. Godoy, H. Tan, M. Bellini, B. J. Allen, J. Jankovic, H. A. Miller, A. C. Alba‐Rubio, D. R. Dekel, *Adv. Funct. Mater.* **2020**, *30*, 2002087.

[5] H. A. Miller, A. Lavacchi, F. Vizza, M. Marelli, F. D. Benedetto, *Angew, Chem, Int, Ed,* **2016**, *55*, 6004.

[6] T. Bhowmik, M. K. Kundu, S. Barman, *ACS Catal,* **2016**, *6*, 1929.

[7] T. Zhao, Y. Hu, M. Gong, R. Lin, S. Deng, Y. Lu, X. Liu, Y. Chen, T. Shen, Y. Hu, L. Han, H. Xin, S. Chen, D. Wang, *Nano Energy* **2020**, *74*, 104877.

[8] H. Yu, E. S. Davydova, U. Ash, H. A. Miller, L. Bonville, D. R. Dekel, R. Maric, *Nano Energy* **2019**, *57*, 820.

[9] H. A. Miller, F. Vizza, M. Marelli, A. Zadick, L. Dubau, M. Chatenet, S. Geiger, S. Cherevko, H. Doan, R. K. Pavlicek, S. Mukerjee, D. R. Dekel, *Nano Energy* **2017**, *33*, 293.

[10] J. Zheng, S. Zhou, S. Gu, B. Xu, Y. Yan, *J. Electrochem. Soc.* **2016**, *163*, F499.

[11] C. Xu, Q. Chen, R. Ding, S. Huang, Y. Zhang, G. Fan, *Chin. J. Catal.* **2021**, *42*, 251.

[12] M. V. Pagliaro, C. Wen, B. Sa, B. Liu, M. Bellini, F. Bartoli, S. Sahoo, R. K. Singh, S. P. Alpay, H. A. Miller, D. R. Dekel, *ACS Catal.* **2022**, *12*, 10894.

[13] A. K. Ipadeola, P. V. Mwonga, S. C. Ray, R. R. Maphanga, K. I. Ozoemena, *ChemElectroChem* **2020**, *7*, 4562.

[14] H. Kim, J. M. Yoo, D. Y. Chung, Y. Kim, M. Jung, M. S. Bootharaju, J. Kim, S. Koo, H. Shin, G. Na, B. S. Mun, J. H. Kwak, Y.-E. Sung, T. Hyeon, *ACS Nano* **2022**, *16*, 16529.

[15] Y. Qiu, L. Xin, Y. Li, I. T. McCrum, F. Guo, T. Ma, Y. Ren, Q. Liu, L. Zhou, S. Gu, M. J. Janik, W. Li, *J. Am. Chem. Soc.* **2018**, *140*, 16580.

[16] A. K. Ipadeola, P. V. Mwonga, K. I. Ozoemena, *Electrochim. Acta* **2021**, *390*, 138860.

[17] S. Kim, S. Yoo, S. Shin, A. A. El‐Zoka, O. Kasian, J. Lim, J. Jeong, C. Scheu, J. Neugebauer, H. Lee, M. Todorova, B. Gault, *Adv. Mater.* **2022**, *34*, 2203030.

[18] H. Liu, J. Fu, H. Li, J. Sun, X. Liu, Y.Qiu, X. Peng, Y. Liu, H. Bao, L. Zhuo, L. Zhuo, R. Cao, S. Zhang, J. Luo, *Appl. Catal. B: Environ.* **2022**, *306*, 121029.

[19] L. Wang, Z. Xu, C. Kuo, J. Peng, F. Hu, L. Li, H. Chen, J. Wang, S. Peng, *Angew. Chem. Int. Ed.* **2023**, *62*, e202311937.

[20] Y. Yang, L. Shi, Q. Liang, Y. Liu, J. Dong, T. Isimjan, B. Wang, X. Yang, *Chin. J. Catal.* **2024**, *56*, 176.

[21] P. Zhao, L. Deng, C. Sun, X. Li, X. Tian, Z. Li, W. Sheng, *ACS Catal.* **2025**, *15*, 1352.

[22] Y. Yuan, X.-Q. Wu, X. Yin, H.-Y. Ruan, Y.-P. Wu, S. Li, G. Hai, G. Zhang, S. Sun, D.-S. Li, *Angew. Chem. Int. Ed*. **2024**, *63*, e202412680.

[23] R. Sgarbi, H. Doan, S. Amigues, N. Bibent, V. Martin, V. Zemtsova, F. Jaouen, J. Thuilliez, M. Chatenet, *ACS Catal,* **2025**, *15*, 9379.
